# Resting-state cortical alpha frequency in Parkinson’s disease and multiple system atrophy: associations with diagnosis, age, and symptom burden

**DOI:** 10.3389/fnagi.2026.1928414

**Published:** 2026-09-30

**Authors:** Daichao Chen, Huada Tang, Yanfei Pan, Hui Sang, Saiya Gu, Xinpeng Wang, Kai Li, Fan Li, Yunchuang Sun, Jing Chen, Wei Sun, Jue Zhang, Zhaoxia Wang, Luhua Wei

**Affiliations:** 1 Department of Neurology, Peking University First Hospital, Beijing, China; 2 Academy for Advanced Interdisciplinary Studies, Peking University, Beijing, China; 3 Department of Neurology, Taiyuan City Central Hospital, Taiyuan, Shanxi, China; 4 The First School of Clinical Medicine, Peking University, Beijing, China; 5 Institute of Medical Technology, Peking University, Beijing, China; 6 School of Mechanics and Engineering Science, Peking University, Beijing, China

**Keywords:** aging, alpha slowing, cortical networks, individual alpha frequency, multiple system atrophy, non-motor symptoms, Parkinson’s disease, resting-state EEG

## Abstract

**Background:**

Parkinson’s disease (PD) and multiple system atrophy (MSA) are clinically overlapping neurodegenerative disorders that present with a complex, heterogeneous burden of motor and non-motor symptoms. Resting-state EEG reveals that cortical alpha slowing is a hallmark of pathological aging, yet its specific presence and clinical significance in these distinct synucleinopathies remain incompletely understood. Therefore, precisely quantifying the individual alpha frequency serves as a critical, non-invasive approach to characterize cortical network dysfunction across synucleinopathies and evaluate how aging moderates its association with clinical symptom burden.

**Methods:**

We analyzed source-reconstructed resting-state EEG data from 104 participants, comprising patients with PD, patients with the parkinsonian (MSA-P) and cerebellar subtypes (MSA-C) of MSA, and healthy controls. To accurately capture oscillatory dynamics, a data-driven masking empirical mode decomposition approach was applied to extract the individual alpha frequency across canonical cortical networks. General linear models tested diagnostic group differences, age-related effects, and age-dependent associations between individual alpha frequency and clinical symptom scores.

**Results:**

Relative to healthy controls, both PD and MSA-C exhibited significant lower cortical IAF, whereas MSA-P showed no significant global IAF difference. At the network level, significant reduction of IAF was detected in the visual and dorsal attention networks in both PD and MSA-C. Advancing age independently contributed to alpha slowing across all participants without accelerating the disease-related trajectories. Notably, chronological age significantly moderated the relationship between alpha slowing and non-motor symptoms. In older patients, reduced individual alpha frequency was strongly associated with a higher burden of overall non-motor, autonomic, and psychiatric/cognitive symptoms, a relationship not observed in younger patients or with motor symptom severity.

**Conclusion:**

Cortical alpha slowing was detected in PD and MSA-C, but not in MSA-P. Age independently contributes to alpha slowing and strengthens its association with non-motor symptom burden, supporting age-aware interpretation of resting-state electroencephalography biomarkers in synucleinopathies.

## 1 Introduction

Parkinson’s disease (PD) and multiple system atrophy (MSA) are clinically overlapping α-synucleinopathies that frequently share motor and non-motor manifestations, yet they differ substantially in their underlying pathology and clinical trajectories (Kalia and Lang, 2015; Poewe et al., 2022; Krismer et al., 2024). Pathologically, PD is primarily driven by neuronal α-synuclein Lewy body accumulation and dopaminergic neuronal loss in the substantia nigra (Poewe et al., 2017). In contrast, MSA is characterized by oligodendroglial cytoplasmic α-synuclein inclusions with prominent striatonigral and olivopontocerebellar degeneration. Based on the predominant motor presentation, MSA is further categorized into parkinsonian (MSA-P) and cerebellar (MSA-C) subtypes (Poewe et al., 2022). Clinically, both PD and MSA exhibit profound heterogeneity, presenting with a complex combination of parkinsonism, autonomic dysfunction, sleep disturbances, and neuropsychiatric or cognitive symptoms. However, MSA generally progresses more aggressively and exhibits a poorer response to dopaminergic therapy compared to PD, often resulting in a worse prognosis and severely affected quality of life (Krismer et al., 2024). Given this substantial clinical overlap, identifying non-invasive physiological biomarkers that capture underlying cortical network dysfunction is crucial for better understanding the divergent disease mechanisms and clinical heterogeneity of these syndromes.

Resting-state EEG is a cost-effective and non-invasive neuroimaging modality that captures millisecond-scale neural dynamics, making it an ideal tool for analyzing cortical network dysfunction in neurodegenerative diseases. Among resting-state EEG features, the alpha rhythm, which is the dominant oscillation in the eyes-closed condition, has been extensively investigated due to its central role in intrinsic cortical dynamics and cognitive processing (Pascucci et al., 2025). In particular, the individual alpha frequency (IAF), typically defined as the dominant or center frequency of the alpha rhythm, is closely associated with a wide range of cognitive abilities, including memory performance (Klimesch et al., 1993), attentional processing (Zhang et al., 2021), reaction time (Surwillo, 1963) and perceptual sensitivity (Samaha and Postle, 2015; Gray and Emmanouil, 2020; Tarasi and Romei, 2024). A slowing of the alpha frequency is commonly observed in healthy aging (Turner et al., 2023; Park et al., 2024) as well as in various neurological disorders, and has been proposed as an objective biomarker to assess cognitive impairments in stroke (Zhao et al., 2025b), isolated rapid eye movement sleep behavior disorder (Schopp et al., 2025), Alzheimer’s disease (Cecchetti et al., 2024), and PD (Geraedts et al., 2018). In PD, for instance, slowing of resting-state EEG has been reported as increased theta-band (4–8 Hz) power, a reduced fast-to-slow power ratio, and a decreased dominant frequency (Stoffers et al., 2007; Jaramillo-Jimenez et al., 2023), with these electrophysiological alterations also linked to cognitive symptom severity (Morita et al., 2011; Geraedts et al., 2018; Zhao et al., 2025a).

Although alpha slowing has been repeatedly reported in PD and other neurodegenerative disorders, whether and how this phenomenon occurs in MSA, as well as its clinical associations, remains incompletely characterized. Preliminary spectral power analyses comparing PD and MSA have suggested that overall spectral slowing is less severe in MSA than in PD (Liu et al., 2023; Wang et al., 2024). However, these studies often pooled MSA-P and MSA-C patients into a single group, a methodological choice that may obscure subtype-specific electrophysiological signatures and mask true pathophysiological differences. Moreover, most previous studies have relied on sensor-level spectral analyses with fixed frequency bands, which may be less sensitive to individualized alpha oscillatory dynamics and their spatial organization across cortical networks. Because IAF is sensitive to the functional state of large-scale cortical networks, we hypothesized that cortical IAF alterations may be present not only in PD but also in MSA, and may relate to a wider range of clinical manifestations beyond cognitive impairment alone. Furthermore, given the distinct topographical distribution of pathology across PD, MSA-P, and MSA-C, we explored whether cortical alpha slowing would show different network-wise patterns across these diagnostic groups. Finally, because healthy aging independently contributes to alpha slowing and may reduce neural compensatory capacity, we further hypothesized that the clinical consequences of this electrophysiological deficit are age-dependent, such that older patients may show a stronger association between alpha slowing and clinical symptom burden.

To test these hypotheses, we analyzed cortical source-level IAF from resting-state EEG in patients with PD, MSA-P, MSA-C, and healthy controls (HC). To accurately capture both the spatial distribution and temporal dynamics of cortical oscillations, we applied source reconstruction with a data-driven masking empirical mode decomposition (EMD) approach to reliably extract the IAF across canonical cortical networks. The primary objectives of this study were threefold: (1) to map and compare global and network-level cortical IAF alterations among PD, MSA-P, and MSA-C; (2) to determine whether chronological age contributes independently to alpha slowing and whether age-related IAF trajectories differ across diagnostic groups; and (3) to investigate whether chronological age moderates the relationship between cortical IAF and clinical symptom burden, encompassing both motor and non-motor domains.

## 2 Materials and methods

### 2.1 Participants

The study cohort comprised a total of 104 participants, including HC and patients diagnosed with PD, MSA-P, and MSA-C. These participants were recruited from the Department of Neurology at Peking University First Hospital between 2022 and 2026. Patient inclusion required: (1) an age range of 40 to 85 years; (2) a diagnosis of clinically established or probable PD based on the 2015 Movement Disorder Society (MDS) criteria (Postuma et al., 2015), or clinically established or probable MSA based on the 2022 MDS criteria (Wenning et al., 2022); and (3) a modified Hoehn and Yahr (H&Y) stage ranging from I to IV. Individuals were excluded if they presented with: (1) cognitive decline, indicated by a Chinese Mini Mental Status (CMMS) (Katzman et al., 1988) score below 24; (2) any history of stroke, epilepsy, or other severe psychiatric or neurological conditions; or (3) severe resting tremor (scoring 4 on any resting tremor item of the MDS Unified Parkinson’s Disease Rating Scale Part III [MDS-UPDRS III]) to reduce motion artifacts during EEG recordings. Diagnoses were independently verified by two senior neurologists. The MSA cohort was further categorized into MSA-P or MSA-C subtypes depending on the predominance of parkinsonian or cerebellar symptoms (Wenning et al., 2022). The study protocol was approved by the Peking University First Hospital Ethics Committee, and all participants provided written informed consent.

### 2.2 Clinical assessments

Motor severity was assessed immediately after EEG acquisition using the MDS-UPDRS III for PD and MSA patients, and the Unified Multiple System Atrophy Rating Scale Part II (UMSARS II) for the MSA cohort. Non-motor symptom burden was evaluated across all patient groups within 3 months of the EEG session using the Non-Motor Symptoms Questionnaire (NMSQ), which yields a total score as well as sub-scores for specific domains including autonomic, sleep, and psychiatric and cognitive symptoms.

### 2.3 EEG recording and preprocessing

Resting-state EEG was recorded for at least 3 min while participants sat in a comfortable chair, remaining awake, relaxed, and as still as possible with their eyes closed. During the recording, an experimenter was present in the room to continuously monitor the patient’s behavior and the live EEG trace. If signs of drowsiness (e.g., slowing of the background rhythm, behavioral signs of sleep) or eye-opening occurred, the recording was paused, and the participant was verbally alerted. All patients were assessed in their off-medication state. Recordings were obtained via a 64-channel Greentek cap (10–10 international system) connected to either a Neurofax EEG-1200 (Nihon Kohden, Tokyo, Japan; *n* = 57) or a PN-NET (Yunshen Technology, Beijing, China; *n* = 47) system. Detailed information about both systems were provided in Supplementary Table S11. Electrode impedances were kept below 10 kΩ. Data were acquired at a sampling rate of 1,000 Hz.

Preprocessing was conducted via custom Python code utilizing the MNE-Python (Gramfort et al., 2013) and MEEGkit packages. Excessively noisy channels and time windows were manually annotated and discarded. The annotation of excessively noisy channels and time windows was performed by a researcher blinded to the participants’ diagnostic group. Signals were downsampled to 256 Hz. Line noise (50 Hz) was removed via the iterative Zapline algorithm (“dss_line_iter” in MEEGkit) (de Cheveigné, 2020). Signals were subsequently re-referenced to the common average (omitting channels that were previously marked as bad). Artifacts were suppressed using an automatic independent component analysis (ICA) pipeline, where components classified as “muscle,” “eye,” “heart,” “channel noise,” or “line noise” with a probability ≥ 0.7 by MNE-ICALabel (Li et al., 2022) were discarded. The cleaned data were then processed by a one-pass, zero-phase FIR band-pass filter (1–80 Hz; transition bands: 1 Hz low, 10 Hz high). Following spherical spline interpolation of the initially excluded channels, the continuous recordings were divided into 8-s, 50%-overlapping epochs. To ensure the accuracy of IAF estimation, participants with the number of retained epochs less than 22 were excluded from analysis (a continuous 3-min clean recording can generate 44 epochs). Two participants in the HC group were thus excluded. For participants with more than 44 epochs, only the first 44 epochs were preserved to ensure data length consistency across the cohort. The final study cohort comprised 102 participants, including HC (*n* = 32), patients with PD (*n* = 28), MSA-P (*n* = 19), and MSA-C (*n* = 23). The distribution of device usage was comparable across the diagnostic groups [HC: 20/12; PD: 16/12; MSA-P: 10/9; MSA-C: 9/14; 𝜒^2^(3) = 3.103, *p* = 0.376], to reduce equipment-driven biases. Nevertheless, because device-related variation remained possible, recording device was included as a categorical fixed-effect covariate in all primary statistical models. Data quality was comparable across groups, with non-parametric Kruskal-Wallis tests revealing no significant inter-group differences in the number of interpolated channels (HC: 3.5 [2–6], median [25th–75th percentile]; PD: 4 [3–6]; MSA-P: 4 [3–5]; MSA-C: 3 [3–6]; *H*(3) = 0.205, *p* = 0.977), rejected ICA components (HC: 3.5 [2–6]; PD: 3.5 [2–6]; MSA-P: 6 [4–8]; MSA-C: 4 [3–6]; *H*(3) = 4.687, *p* = 0.196), the number of retained epochs (HC: 43 [40–44]; PD: 44 [42–44]; MSA-P: 44 [39–44]; MSA-C: 44 [43–44]; *H*(3) = 4.488, *p* = 0.213), or the final recording duration covered by these epochs in seconds (HC: 180 [168–180]; PD: 180 [172–180]; MSA-P: 180 [164–180]; MSA-C: 180 [176–184]; *H*(3) = 3.798, *p* = 0.284). The detailed information about these quality control metrics of all participants were provided in Supplementary Table S12.

### 2.4 Source reconstruction and cortical parcellation

Cortical electrophysiological activity was estimated using source reconstruction in MNE-Python (Gramfort et al., 2013). We utilized a source space consisting of 8,196 dipoles defined on the FreeSurfer fsaverage template (Fischl et al., 1999) via a recursively subdivided octahedron (oct6). The forward solution was generated based on standard electrode coordinates and a three-compartment boundary element model (skin, outer skull, and inner skull) derived from the template.

Because preprocessing steps can introduce rank deficiency, we explicitly estimated the data rank prior to computing the noise covariance matrix. To guarantee a well-conditioned and stable matrix, the Oracle Approximating Shrinkage (OAS) technique was applied (Chen et al., 2010). Source inversion was achieved using the exact low-resolution brain electromagnetic tomography (eLORETA) method (Pascual-Marqui et al., 2011). For the inverse operator, we applied a loose orientation constraint (loose = 0.8) and only the normal direction to the cortical surface was kept for the source estimates. A signal-to-noise ratio of 1.0 was chosen for regularization.

The resulting cortical dipoles were grouped into 100 regions of interest (ROIs) utilizing the 7-network Schaefer atlas (Schaefer et al., 2018), which categorizes regions into the default mode (DMN), somatomotor (SMN), visual (VN), salience/ventral attention (SVAN), dorsal attention (DAN), limbic (LN), and frontoparietal (FPN) networks (Figure 1A). The sign-flip averaging method was used to derive the time series for each ROI.

**Figure 1:**
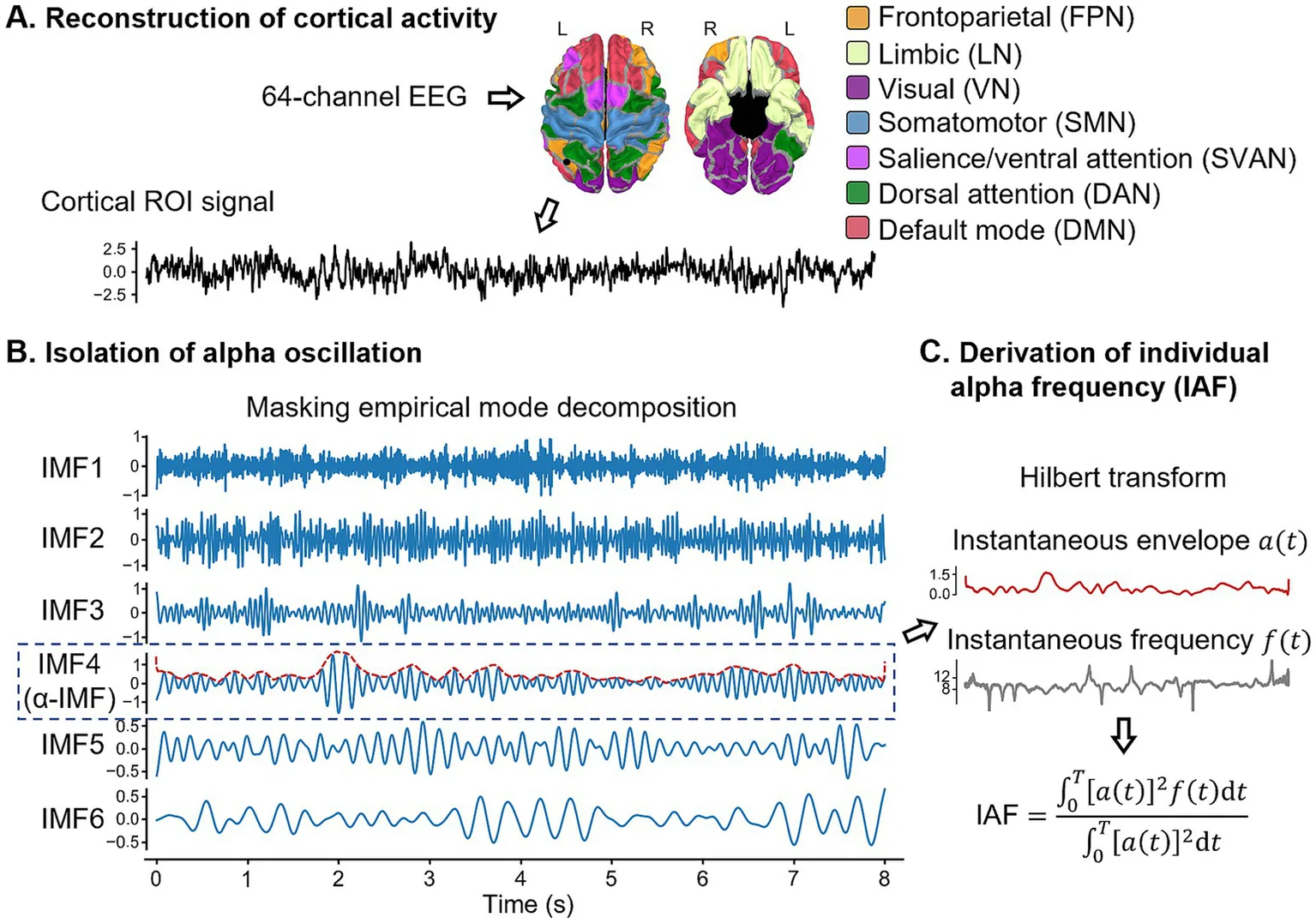
Methodological pipeline for the derivation of individual alpha frequency (IAF). **(A)** Reconstruction of cortical activity. Sensor-level 64-channel EEG data are processed by source reconstruction to estimate activity within cortical regions of interest (ROIs). These ROIs are divided according to the Schaefer’s 100-parcellation atlas (7-network version). **(B)** Isolation of alpha oscillation. The reconstructed cortical signals undergo masking empirical mode decomposition (EMD), which separates the signal into distinct intrinsic mode functions (IMFs 1–6). IMF4 possesses a center frequency aligning with the alpha band (8–13 Hz) and is thus designated as α-IMF. **(C)** Derivation of IAF. The Hilbert transform is applied to α-IMF to extract its instantaneous envelope *a*(*t*) and instantaneous frequency *f*(*t*). The final IAF value is calculated as the power-weighted average of the instantaneous frequency over the time window of duration *T*. DAN, dorsal attention network; DMN, default mode network; FPN, frontoparietal network; LN, limbic network; SMN, somatomotor network; SVAN, salience/ventral attention network; VN, visual network.

### 2.5 Extraction of individual alpha frequency

Given the non-stationary and non-linear properties of EEG, we here used a data-driven method, EMD (Huang et al., 1998), to isolate the alpha oscillation and derive the individual alpha frequency (IAF) (Figures 1B,C). EMD adaptively decomposes signals into several intrinsic mode functions (IMFs), ranging from fast to slow oscillations. However, the original EMD algorithm is prone to mode mixing due to signal intermittency, where a single alpha oscillation might split across several IMFs (Supplementary Figure S1A, left). To overcome this limitation and ensure reliable isolation of the alpha oscillation, we used Masking EMD (Deering and Kaiser, 2005; Opie et al., 2024), which introduces sinusoidal masking signals to guide the decomposition process and can effectively mitigate mode mixing (Supplementary Figure S1A, right). The frequencies of the masking signals were set to be 65, 25, 16, 10, 6, and 3 Hz, leading masking EMD to consistently produce six IMFs (IMF1–IMF6) that were centered around these frequencies. The Hilbert transform was applied to the IMFs, yielding instantaneous envelope $a_{i}(t)$ and instantaneous phase $\theta_{i}(t)$ (and thus instantaneous frequency $f_{i}(t)=\theta_{i}^{\prime }(t)/(2\pi )$ ) for each IMF i .

To visualize the distribution of instantaneous frequencies, we computed the marginal Hilbert spectrum. For the whole signal with duration T , its marginal Hilbert spectrum h(f) (Huang et al., 1998) is formally defined by


$$h(f)=\frac{1}{T}\sum_{i}\int_{0}^{T}a_{i}^{2}(t)\delta [f-f_{i}(t)]dt.$$

In the discretized form, the marginal Hilbert spectrum is derived by dividing the frequency axis into bins and accumulating the instantaneous power $a_{i}^{2}(t)$ with instantaneous frequency $f_{i}(t)$ located in each bin, which is equivalent to a power-weighted histogram of instantaneous frequency. For each cortical ROI from each participant, the marginal Hilbert spectra were averaged across all 8-s epochs. Since the absolute signal power differed among brain regions and participants, the marginal Hilbert spectrum was further normalized by the total power over the full frequency range as follows, such that the area under the curve of $h_{n}(f)$ sums to 1.


$$h_{n}(f)=\frac{h(f)}{\int_{f}h(f)df}=\frac{h(f)}{T^{-1}\sum_{i}\int_{0}^{T}a_{i}^{2}(t)dt}.$$

Likewise, for the i -th IMF, we can also compute its marginal Hilbert spectrum by $h^{\left(i\right)}(f)=T^{-1}\int_{0}^{T}a_{i}^{2}(t)\delta [f-f_{i}(t)]dt$ , and normalize it by the total signal power $\int_{f}h(f)df$ . The marginal Hilbert spectrum of individual IMFs showed that the IMFs generated from masking EMD were clearly separated in their frequency distribution, and spectral lines of IMFs of the same order concentrated into a narrow frequency range (Supplementary Figures S1B, S2).

To further investigated which IMF corresponds to alpha oscillation, we calculated the center frequency for each IMF. The center frequency of IMF i was computed by the power-weighted average of its instantaneous frequency in the time domain (which is also equal to the center of gravity of its marginal Hilbert spectrum in the frequency domain),


$${\bar{f}}_{i}=\frac{\int_{0}^{T}{\left[a_{i}(t)\right]}^{2}f_{i}(t)dt}{\int_{0}^{T}{\left[a_{i}(t)\right]}^{2}dt}.$$

Center frequencies were then averaged across all epochs within each ROI. The center frequencies of the IMF1–IMF6 were 81.395 ± 1.380, 30.596 ± 1.791, 15.070 ± 0.739, 9.404 ± 0.287, 5.854 ± 0.103, and 3.158 ± 0.046 Hz (Mean ± SD across 102 participants ×100 ROIs), respectively (Supplementary Figure S2). Since the marginal Hilbert spectrum and the center frequency of IMF4 consistently aligned well with the canonical alpha band (8–13 Hz), IMF4 is designated as the α-IMF, with its center frequency identified as IAF.

Network-level IAFs were derived by averaging the regional IAF values within each of the seven canonical networks, and a global cortical mean IAF was calculated by averaging across all 100 ROIs.

### 2.6 Statistical analysis

Statistical analyses were performed in Python using the Scipy (Virtanen et al., 2020) and Statsmodels packages (Seabold and Perktold, 2010). To evaluate the main effects of clinical diagnosis (group) and chronological age on IAF, a single general linear model (GLM) was employed across the entire cohort, including group, age, gender, and recording device as predictors. This GLM was evaluated first using the global cortical mean IAF (Supplementary Table S1), and subsequently repeated for the IAF of each of the seven cortical networks (Supplementary Table S4). Where a significant main effect of group was observed, *post hoc* pairwise comparisons of covariate-adjusted group means were conducted using Student’s *t*-tests (Supplementary Tables S2, S5). To examine whether the diagnostic group effects on IAF varied across cortical networks, we fitted the network-level IAF using a linear mixed-effects model including group, network, the group × network interaction, age, gender, and recording device as fixed effects, with a participant-specific random intercept (Supplementary Table S6). To determine whether diagnostic effects differed between recording systems, we fitted an additional model containing a group × device interaction (Supplementary Table S3A) and performed pairwise comparisons among groups within the cohort of each recording system (Supplementary Table S3B).

To further quantify the continuous relationship between age and cortical mean IAF, partial correlation coefficients (*r_p_*) were derived from the GLM *t*-statistics, adjusting for the same covariates. Additionally, to investigate whether the trajectories of the age-IAF relationship were parallel across the different clinical groups, an age × group interaction term was tested in a separate, supplementary GLM (Supplementary Table S7).

To investigate the clinical relevance of IAF, we next assessed its relationship with motor (MDS-UPDRS III) and non-motor (NMSQ) symptom scores within the patient cohort. Because healthy aging and neurodegeneration independently contribute to alpha slowing, we hypothesized that older patients may have diminished compensatory capacity. Consequently, the relationship between electrophysiological alterations and clinical symptoms might be age-dependent. Before pooling the patient groups for clinical analyses, we formally tested diagnostic heterogeneity in the age-dependent symptom–IAF association. For each motor and non-motor symptom subdomain score, a full factorial GLM included group, age, symptom score, group × age, age × symptom score, group × symptom, group × age × symptom score interaction, gender, and recording device (Supplementary Table S8A). The omnibus three-way interaction tested whether age moderation differed among PD, MSA-P, and MSA-C. When the group × age × symptom score interaction was not significant, this term was removed, and the reduced model was evaluated for group × age and group × symptom score interactions to test for disease-specific baseline slopes (Supplementary Table S8B). If these disease-specific interaction terms were not statistically significant, they were also removed. The final GLM included group, age, symptom score, the age × symptom score interaction, gender, and recording device as predictors, estimating a transdiagnostic coefficient across PD, MSA-P, and MSA-C while controlling for baseline group differences in IAF. The models were applied at both the global cortical level (Supplementary Table S9) and across the seven spatial networks (Supplementary Table S10). Where interaction terms were not statistically significant, reduced models (excluding the interaction term) were subsequently evaluated to test for direct main effects of symptom scores on IAF.

In the above GLM analysis, categorical predictors (e.g., group, gender, and recording device) were defined using sum coding (deviation coding), and omnibus *F*-statistics were calculated using Type III sums of squares. To quantify the magnitude of the observed statistical effects, effect sizes were reported as partial eta squared (Partial *η*^2^) for main effects and interactions within the GLMs, and as Cohen’s *d* for *post hoc* pairwise comparisons. To rigorously control for Type I errors, the Benjamini-Hochberg False Discovery Rate (FDR) procedure was utilized to correct *p*-values for all analyses involving multiple comparisons. Specifically, FDR correction was applied separately to: (1) the six post-hoc pairwise group comparisons of cortical global IAF (Supplementary Table S2); (2) the six post-hoc pairwise group comparisons of cortical global IAF within the cohort of each device, in the device-stratified analysis (Supplementary Table S3B); (3) the main effect of group on network-level IAF across the seven cortical networks (Supplementary Table S4); (4) the six post-hoc pairwise group comparisons of network-level IAF within each cortical networks (Supplementary Table S5); (5) the group × age × symptom score interaction across the 4 NMSQ subdomains and across the 5 MDS-UPDRS III subdomains (Supplementary Table S8A); (6) the group × age and the group × symptom score interactions across the 4 NMSQ subdomains and across the 5 MDS-UPDRS III subdomains (Supplementary Table S8B); (7) the age × symptom score interaction across the 4 NMSQ subdomains and across the 5 MDS-UPDRS III subdomains (Supplementary Table S9); (8) the 28 network × symptom score interaction models (7 networks × 4 non-motor symptom scores; Supplementary Table S10); and (9) post-hoc pairwise group comparisons of clinical variables that revealed significant differences by the Kruskal-Wallis test (Table 1).

**Table 1:** Demographic and clinical profiles of healthy controls (HC) and patients with Parkinson’s disease (PD) and multiple system atrophy (MSA).

Variable	HC (*n* = 32)	PD (*n* = 28)	MSA-P (*n* = 19)	MSA-C (*n* = 23)	Overall *P*
Age (y)	65.1 (62.0–72.5)	68.2 (65.4–72.3)	67.7 (60.7–71.0)	63.8 (56.4–68.3)	0.337
Gender	22 M/10 F	16 M/12 F	7 M/12 F	14 M/9 F	0.165
Disease duration (y)	–	4.8 (3.0–6.4) ^a^	2.4 (1.3–4.4)	1.7 (1.3–3.0)	**0.001
MDS-UPDRSIII	–	32.0 (24.0–38.0) (*n* = 26)	42.5 (35.0–57.0)^a,b^ (*n* = 18)	33.0 (26.0–37.0)	**0.002
UMSARSII	–	–	21.0 (18.0–25.0) (*n* = 18)	16.0 (15.0–22.0)	0.078
NMSQ	–	16.0 (7.0–21.0) (*n* = 23)	17.0 (14.0–20.0) (*n* = 16)	15.0 (10.0–22.0) (*n* = 18)	0.595

Continuous variables are expressed as median (25th–75th percentile). Age and gender were documented at the time of EEG recording. Disease duration indicates the time elapsed since the initial onset of motor symptoms. For across-group comparisons, the Kruskal–Wallis test was applied to continuous variables, and the Chi-square test was used for gender. Because this table provides descriptive information, overall *p*-values were not corrected for multiple comparisons. For rows with an overall *p* < 0.05, *post hoc* pairwise analyses were conducted using Dunn’s test with False Discovery Rate (FDR) correction. Superscripts denote statistical significance (FDR-corrected *p* < 0.01) for pairwise comparisons: ᵃsignificantly different from MSA-C; ᵇsignificantly different from PD. Because certain clinical assessments were integrated into the study protocol after initial recruitment had begun, scores are missing for some participants (13 for NMSQ, 3 for MDS-UPDRS III, and 1 for UMSARS II). HC, healthy controls; MDS-UPDRS III, Movement Disorder Society Unified Parkinson’s Disease Rating Scale part III; NMSQ, Non-Motor Symptoms Questionnaire; UMSARS II, Unified Multiple System Atrophy Rating Scale Part II.

## 3 Results

### 3.1 Demographic and clinical characteristics

The demographic and clinical profiles of the 104 participants are detailed in Table 1. The four groups (HC, PD, MSA-P, and MSA-C) were comparable in terms of age [*H*(3) = 3.376, *p* = 0.337, by Kruskal–Wallis test] and gender distribution [𝜒^2^(3) = 5.090, *p* = 0.165, by Chi-squared test]. Disease duration varied significantly among the patient groups [*H*(2) = 13.114, *p* = 0.001], being longest in the PD cohort. Motor symptom severity, assessed via MDS-UPDRS III, also showed significant differences among groups [*H*(2) = 12.181, *p* = 0.002], where the MSA-P group demonstrated the highest MDS-UPDRS III scores. In contrast, overall non-motor symptom burden, assessed by the total NMSQ score, was comparable across the three patient groups [*H*(2) = 1.039, *p* = 0.595].

### 3.2 Cortical global and network-level IAF alterations in PD and MSA

We first investigated whether global and network-level cortical IAF differed across PD, MSA subtypes, and HCs. The cortical mean marginal Hilbert spectrum showed a visible leftward shift of the instantaneous frequency distribution in the alpha band in PD and MSA-C compared to HC (Figure 2A). Statistical modeling of cortical IAF supported this observation. At the global level, the GLM revealed a significant main effect of diagnosis group on mean cortical IAF after adjusting for age, gender, and recording device [*F*(3, 95) = 3.594, *p* = 0.016, Partial *η*^2^ = 0.102; Supplementary Table S1A]. *Post hoc* pairwise comparisons demonstrated that both PD and MSA-C patients exhibited significantly lower cortical mean IAF compared to HC (PD vs. HC: adjusted mean difference = −0.156 Hz, 95% CI [−0.271, −0.041], *t*(95) = −2.692, FDR-corrected *p* = 0.044, Cohen’s *d* = −0.700; MSA-C vs. HC: adjusted mean difference = −0.156 Hz, 95% CI [−0.280, −0.031], *t*(95) = −2.486, FDR-corrected *p* = 0.044, Cohen’s *d* = −0.698) (Figure 2B and Supplementary Table S2). In contrast, cortical mean IAF in MSA-P did not differ significantly from that in HC (adjusted mean difference = −0.030 Hz, 95% CI [−0.161, 0.102], *t*(95) = −0.452, FDR-corrected *p* = 0.783, Cohen’s *d* = −0.135). The direct comparison between MSA-C and MSA-P was also not statistically significant (adjusted mean difference = −0.126 Hz, 95% CI [−0.267, 0.016], *t*(95) = −1.761, FDR-corrected *p* = 0.122, Cohen’s *d* = −0.564). Thus, although lower global IAF was detected in MSA-C but not MSA-P relative to HC, the present analysis did not establish a statistically significant difference between the MSA subtypes.

**Figure 2:**
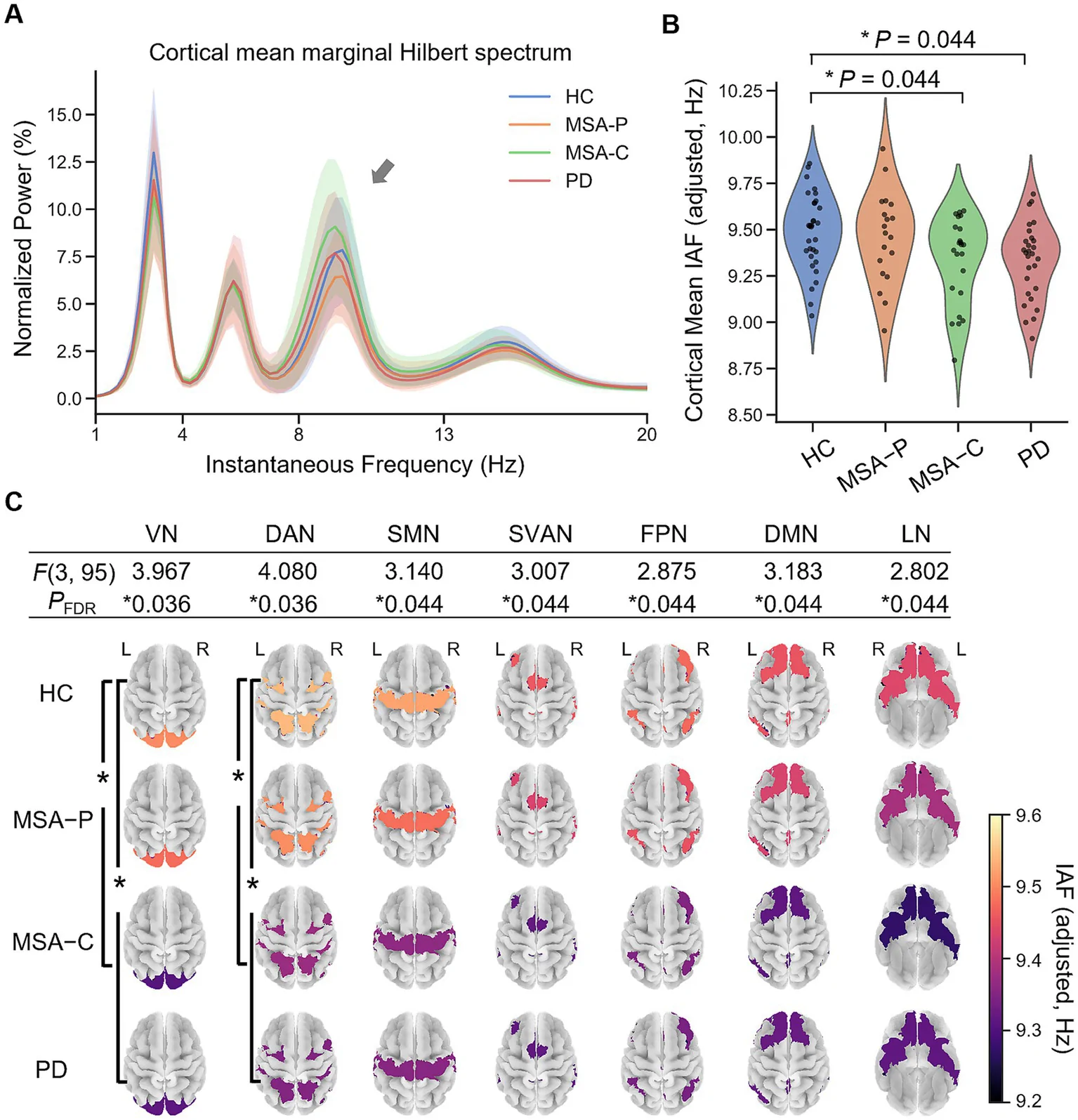
Group-level comparisons of cortical IAF among HC, PD and MSA. **(A)** Comparisons of cortical mean marginal Hilbert spectrum. For each cortical ROI of each participant, the marginal Hilbert spectrum were normalized by the total power over the whole frequency range, such that the area under the curve sums to 100%. The normalized marginal Hilbert spectra of all cortical ROIs were averaged within participants. Solid lines represent the group mean, and shaded regions denote the SD across participants (*n* = 102). The gray arrow indicates the spectral peak contributed by alpha oscillation. **(B)** Comparisons of cortical mean IAF among groups. The plotted adjusted IAF values represent partial residuals derived from the general linear model (GLM) regressing out the effects of covariates (age, gender, and recording device). **(C)** Network-level comparisons of cortical IAFs among groups. The reported *F*-statistics and FDR-corrected *p*-values represent the main effect of group for each cortical network derived from the GLM. For cortical mean IAF and network IAFs, *post hoc* pairwise comparisons between groups were conducted using Student’s *t*-tests with FDR correction. *, FDR-corrected *p* < 0.05.

Interestingly, recording device was associated with cortical mean IAF [*F*(1, 95) = 5.877, *p* = 0.017, Partial *η*^2^ = 0.058, Supplementary Table S1A]. To examine whether group effects varied by recording device, we additionally tested the group×device interaction. This interaction was not significant [*F*(3, 92) = 0.590, *p* = 0.623, Partial *η*^2^ = 0.019, Supplementary Table S3A], providing no evidence that diagnostic group contrasts differed between systems. Device-stratified estimates were directionally consistent for the PD–HC and MSA-C–HC contrasts, although none survived FDR correction (Supplementary Table S3B).

At the network level, these IAF alterations exhibited a widespread spatial distribution (Figure 2C). Separate GLMs showed significant main effects of group across all seven canonical networks (all FDR-corrected *p* < 0.05; Supplementary Table S4). *Post hoc* comparisons revealed that reduced IAF in PD and MSA-C compared to HC was observed in all networks, but the differences were significant only in the VN and DAN after FDR correction (Supplementary Table S5). A linear mixed-effects model testing the group × network interaction did not show a statistically significant interaction [𝜒^2^(18) = 27.255, *p* = 0.074; Supplementary Table S6], indicating that the present data provided no evidence that the magnitude of diagnostic group differences in IAF varied across networks. Together, these results demonstrate that global cortical IAF was reduced in PD and MSA-C relative to healthy controls, without statistically divergent spatial profiles.

### 3.3 Advancing age is associated with cortical alpha slowing across all groups

Independent of clinical diagnosis, advancing age was strongly associated with a slowing of the alpha rhythm. Across the entire cohort, age exhibited a significant negative partial correlation with cortical mean IAF (*β* = −0.008 (Hz/year), 95% CI [−0.014, −0.002], *t*(95) = −2.833, *r_p_* = −0.279, *p* = 0.006; Figure 3A and Supplementary Table S1B), after adjusting for covariates (group, gender, and recording device). To determine whether the neurodegenerative disease process accelerated this age-related slowing, we further evaluated the group × age interaction. This interaction was not statistically significant [*F*(3, 92) = 1.564, *p* = 0.203, Partial *η*^2^ = 0.049; Supplementary Table S7]. Thus, we found no evidence to suggest that the rate of age-related alpha slowing differed across the diagnostic groups, which justifies fitting a common age-related slope across all participants (Figure 3B).

**Figure 3:**
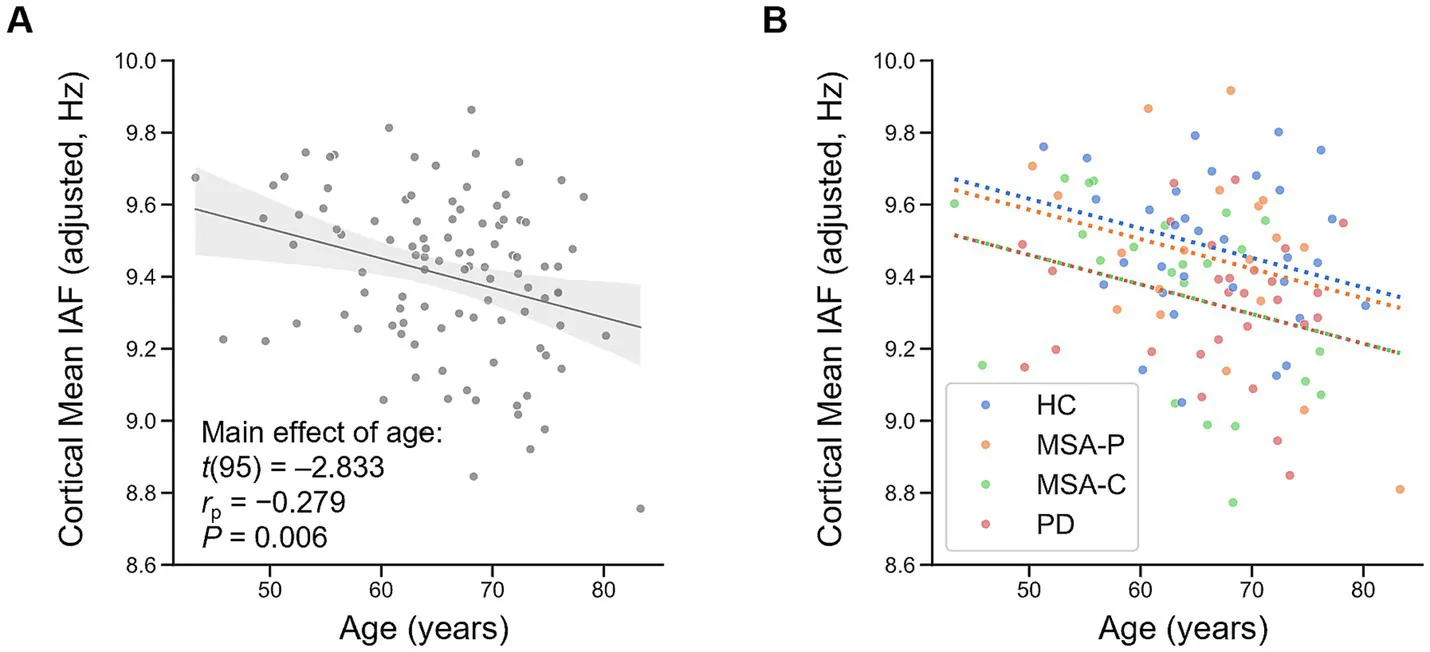
Main effects of aging on the IAF. **(A)** Partial correlation illustrating the relationship between the age and the cortical mean IAF across all participants. Plotted IAF values on the *y*-axis represent partial residuals adjusted for group, gender and recording device. Partial correlation coefficients (*r*_p_) and *p*-values were derived from GLM analysis. **(B)** The relationship between the age and the IAF in each group. Because group × age interaction was not statistically significant, the GLM fits a common slope across all groups, resulting in parallel regression lines (indicated by dashed lines). Plotted IAF values are adjusted for gender and device to isolate the effect of age and group.

### 3.4 Age moderates the association between IAF and non-motor symptoms

Having established that both diagnostic group and chronological aging were associated with alpha slowing, we next investigated how changes in cortical IAF relate to clinical symptom burden. To test whether the clinical relevance of alpha slowing is age-dependent, we utilized an interaction model. Before estimating pooled clinical associations, we formally tested whether the symptom–IAF relationships differed among PD, MSA-P, and MSA-C. The omnibus group × age × symptom interactions were not significant for any NMSQ domain (all FDR-corrected *p* > 0.05; Supplementary S8A), and subsequent reduced models also revealed no significant group × age or group × symptom interactions (all FDR-corrected *p* > 0.05; Supplementary Table S8B). Because there was no statistically detectable evidence of disease-specific slopes, these terms were removed, yielding the final GLM which estimated a transdiagnostic age × symptom effect using the pooled PD, MSA-P, and MSA-C patient cohort. In the pooled cohort, we found that chronological age significantly moderated the relationship between non-motor symptom burden and cortical mean IAF (Supplementary Table S9). Specifically, the age × symptom score interaction was significant for the NMSQ total score [*β* = −0.0017, 95% CI [−0.0026, −0.0008], *t*(49) = −3.658, FDR-corrected *p* = 0.002, Partial *η*^2^ = 0.215], indicating that advancing age strengthened the negative association between overall non-motor symptom burden and IAF. Significant age-dependent effects were also observed for autonomic symptoms (*β* = −0.0034, 95% CI [−0.0056, −0.0012], *t*(49) = −3.084, FDR-corrected *p* = 0.007, Partial *η*^2^ = 0.163) and psychiatric/cognitive symptoms (*β* = −0.0049, 95% CI [−0.0089, −0.0009], *t*(49) = −2.486, FDR-corrected *p* = 0.022, Partial *η*^2^ = 0.112). In contrast, the age × sleep symptom interaction was not significant (*β* = −0.0023, 95% CI [−0.0050, 0.0005], *t*(49) = −1.673, FDR-corrected *p* = 0.101, Partial *η*^2^ = 0.054). To visualize this interaction, the patient cohort was split at the median age of 67.0 years. In the older subgroup, higher total NMSQ scores were strongly correlated with lower IAF (*r_p_* = −0.629, *p* < 0.001; Figure 4A). Similar negative associations were observed for autonomic and psychiatric/cognitive symptoms. Conversely, these relationships were not significant in the younger subgroup.

**Figure 4:**
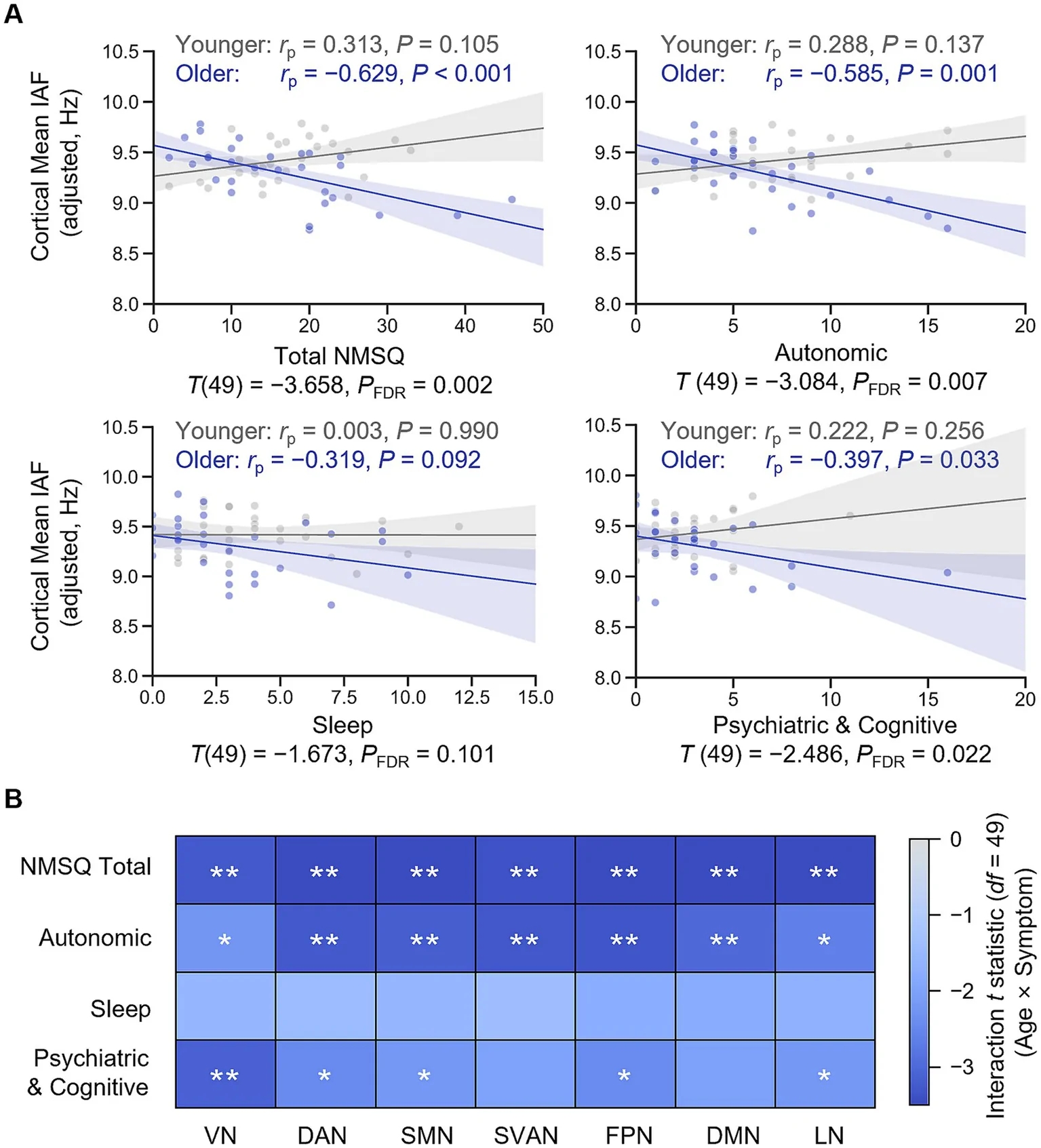
Age-dependent associations between cortical IAF and non-motor symptom severity. **(A)** Scatter plots illustrating the interaction between age and NMSQ scores (total, autonomic, sleep, and psychiatric and cognitive) on cortical mean IAF. To visualize the interaction effect, the patient cohort was divided into “Younger” and “Older” subgroups based on the median age (67.0 years). Solid lines represent linear regression fits, with shaded regions indicating 95% confidence intervals. IAF values shown on the y-axis are adjusted for group, gender, and recording device. The reported *T* statistics and FDR-corrected *p* values were derived from a GLM evaluating the age × symptom interaction across all patients. A negative *T* statistic indicates that advancing age strengthens the negative relationship between symptom severity and IAF. *Post hoc* partial correlation coefficient (*r_p_*) and *p*-values are provided to illustrate the divergent symptom-IAF associations within the younger and older subgroups. **(B)** Heatmap detailing the spatial distribution of the age × symptom interaction effect across all the seven canonical cortical networks. *, FDR-corrected *p* < 0.05; **, FDR-corrected *p* < 0.01.

At the network level, this age-dependent association between non-motor symptom burden and IAF was spatially widespread, with significant age × symptom interactions across nearly all canonical cortical networks, particularly for total NMSQ and autonomic dysfunction (Figure 4B and Supplementary Table S10). Psychiatric and cognitive symptoms showed significant effects in the VN, DAN, SMN, FPN, and LN, whereas the SVAN and DMN did not survive FDR correction.

This age-dependent electrophysiological relationship was detected for non-motor symptoms, but not for motor symptoms. The identical GLMs yielded no significant group × age × symptom score, group × symptom score, or age × symptom score across any MDS-UPDRS III total and subdomain scores, including bradykinesia, rigidity, midline, and tremor scores (all FDR-corrected *p* > 0.05; Supplementary Tables S8, S9). To ensure that direct symptom–IAF associations were not overlooked, we subsequently tested reduced GLMs excluding the interaction terms for variables that showed no significant age-moderation, including NMSQ Sleep and all MDS-UPDRS III domains. These reduced models confirmed that there were also no significant main effects of sleep disturbance or motor severity on mean cortical IAF (all *p* > 0.05). Together, these findings suggest that age-dependent IAF slowing is preferentially associated with broader non-motor, particularly autonomic and psychiatric/cognitive, symptom burden.

## 4 Discussion

In this study, we investigated cortical alpha slowing in PD and MSA using source-level IAF derived from resting-state EEG. We found that cortical global IAF was significantly reduced in PD and MSA-C compared to HCs, and network-level analysis detected significant reductions in the visual and dorsal attention networks in both PD and MSA-C. In contrast, MSA-P did not show significant global IAF slowing relative to controls. In addition, while aging slowed alpha oscillation across all groups, it specifically moderated the relationship between cortical IAF and non-motor symptoms in patients. Specifically, older patients showed stronger negative associations between cortical IAF and non-motor symptom burden, particularly autonomic and psychiatric/cognitive symptoms. These findings suggest that cortical alpha slowing is a network-level electrophysiological feature of both PD and MSA, with potentially heterogeneous expression and age-dependent clinical significance.

### 4.1 Cortical alpha slowing across PD and MSA subtypes

The finding of reduced cortical IAF in PD is consistent with previous resting-state EEG and MEG studies showing that PD is associated with a slowing of spontaneous brain activity. Prior studies have reported increased theta activity, reduced alpha or beta activity, reduced dominant frequency, and reduced fast-to-slow spectral ratios in PD, particularly in association with cognitive impairment (Soikkeli et al., 1991; Stoffers et al., 2007; Morita et al., 2011; Geraedts et al., 2018; Jaramillo-Jimenez et al., 2023). Our results extend this literature in two ways. First, rather than relying on fixed-band spectral power at the sensor level, we quantified IAF using a source-level, data-driven approach designed to capture individual alpha dynamics, providing a complementary view on the alpha slowing phenomena. Second, by mapping IAF across canonical cortical networks, we showed that PD-related slowing was detected primarily in posterior visual-attentional systems. This topography is biologically and clinically plausible, given that the eyes-closed alpha rhythm is typically strongest over the posterior cortex and is closely linked to visual sensitivity and attentional processing (Samaha and Postle, 2015; Gray and Emmanouil, 2020; Zhang et al., 2021; Tarasi and Romei, 2024). These posterior network alterations may be relevant to the abnormal visuospatial processing and visual hallucinations that are frequently observed in PD (Eichelberger et al., 2017; Dauwan et al., 2019), although these specific symptoms were not directly assessed in the present study. Because alpha activity arises from distributed cortical and thalamocortical systems, its slowing in PD should not be attributed to nigrostriatal degeneration. Instead, it may reflect broader extranigral involvement of cortical, thalamic, cholinergic, and brainstem networks associated with neuronal Lewy pathology (Braak et al., 2003; Halgren et al., 2019).

A key novel observation of the present study is that cortical alpha slowing was also evident in MSA-C, whereas no significant alpha slowing was detected in MSA-P. Importantly, the direct statistical comparison between the two MSA subtypes did not reach significance. Therefore, these data do not establish a definitive electrophysiological distinction between the two subtypes. Nonetheless, the differential detectability of alpha slowing relative to controls may help reconcile our findings with previous spectral studies suggesting that overall EEG slowing is less pronounced in pooled MSA cohorts than in PD (Liu et al., 2023; Wang et al., 2024). Because MSA-P and MSA-C differ in their predominant clinical manifestations and underlying patterns of neurodegeneration, combining them into a single MSA group may obscure potentially heterogeneous electrophysiological alterations. Moreover, since PD and MSA have distinct pathological features, the detected alpha slowing in MSA-C might be associated with partly different distributed network mechanisms from PD.

At the network level, alpha slowing in MSA-C was significant in the visual and dorsal attention networks. The mechanisms driving this topographical pattern are likely multifaceted. Structural changes in the visual cortex in MSA-C, including atrophy of the fusiform and lingual gyri, have been reported using MRI (Lin et al., 2020). In addition, the cerebellum connects to the cortical visual attention system through the cortico-cerebellar loop (Buckner et al., 2011; Brissenden et al., 2018), and cerebellar activity has been associated with posterior alpha activity in EEG (Mantini et al., 2007; Liu et al., 2012). Therefore, both degeneration of the visual region and disruption of the cortico-cerebellar circuits caused by olivopontocerebellar degeneration (Ren et al., 2018) may contribute to the alpha slowing observed in the visual and dorsal attention networks. Clinically, these posterior network alterations could be relevant to the dysfunction of visual-attentional, oculomotor, and multisensory integration required for cerebellar gait control in MSA-C. In contrast, MSA-P was characterized by greater parkinsonian motor severity in our cohort but did not show significant global IAF slowing relative to controls. Together with the absence of motor severity–IAF associations, this pattern suggests that IAF might not be an electrophysiological signature for parkinsonian motor burden. However, these interpretations remain speculative because cerebellar, oculomotor, and visuospatial functions were not assessed in detail, and non-motor symptom burden did not differ significantly between the MSA phenotypes.

### 4.2 Aging and the age-dependent clinical relevance of alpha slowing

The association between chronological age and cortical IAF agrees with the broader literature showing that alpha peak frequency declines with aging (Turner et al., 2023; Park et al., 2024). Notably, the absence of a significant group × age interaction suggests that the disease-related shifts in IAF observed in PD and MSA-C are superimposed upon, rather than fundamentally accelerating, the normal age-related trajectory. This parallel decline underscores that age adjustment is essential when evaluating IAF as a neurophysiological biomarker.

The clinical analyses further revealed that aging strengthens the relationship between IAF and non-motor symptom burden. The inference was based on a continuous age × symptom interaction model; the median-age split was used only for visualization and should not be interpreted as evidence for a discrete age threshold. This age-dependent relationship is consistent with a brain reserve framework (Stern, 2012; Stern et al., 2020), in which younger patients may tolerate a degree of cortical oscillatory slowing without major clinical expression, whereas older patients may have reduced compensatory capacity and therefore show tighter coupling between electrophysiological dysfunction and symptom burden. In this context, IAF slowing may not simply reflect chronological aging or diagnostic category alone, but rather an interaction between disease-related cortical network dysfunction and an age-dependent reduction in physiological reserve.

The domain specificity of these clinical associations further illuminates the functional role of the alpha rhythm. The age-dependent link between IAF and psychiatric/cognitive symptoms in PD and MSA extends prior evidence linking slower alpha frequency to cognitive impairment in PD (Morita et al., 2011; Geraedts et al., 2018; Zhao et al., 2025a). Furthermore, the association with autonomic symptoms is particularly relevant for synucleinopathies. Autonomic dysfunction is a core feature of MSA and is also common in PD, reflecting the involvement of peripheral autonomic pathways, brainstem nuclei, and central autonomic networks (Chen et al., 2020; Luo et al., 2025). Cortical IAF does not directly measure peripheral autonomic degeneration or brainstem pathology. Nevertheless, slower alpha dynamics may indicate altered function of distributed cortical systems that interact with autonomic regulation, including limbic, salience, prefrontal, and attentional networks, resulting in the widespread network-level age × autonomic symptom interactions observed in this study.

### 4.3 Methodological considerations and limitations

A methodological strength of this study is the combination of cortical source reconstruction with masking EMD for individualized alpha-frequency estimation. EMD (Huang et al., 1998) provides a data-driven decomposition of non-stationary EEG signals, accounting for the inter-individual variability in their intrinsic alpha oscillation (Haegens et al., 2014). The use of the masking version of EMD (Deering and Kaiser, 2005) reduces mode mixing, allowing for the reliable isolation of the individual alpha oscillatory components. By applying this approach to source-reconstructed cortical signals and summarizing IAF across canonical networks, we were able to examine both global and spatially organized alpha slowing in PD and MSA.

Several limitations should be acknowledged. First, the study was cross-sectional, preventing conclusions about longitudinal IAF decline or the prediction of future clinical progression. Second, although the overall cohort was clinically well characterized, the MSA-P and MSA-C subgroup sizes were modest, which may limit statistical power for direct subtype comparisons and interaction analyses. Third, source reconstruction was performed using template anatomy rather than individual MRI, so the spatial localization of network-level effects should be interpreted as approximate, particularly for inferior and medial regions, where source estimation from scalp EEG is less precise. Forth, despite reassuring sensitivity analyses, residual hardware-related variation from the use of two EEG recording systems cannot be completely excluded. Fifth, non-motor symptoms were assessed using the NMSQ. While this tool is well-validated for PD and suitable for broad screening, it lacks formal psychometric validation for MSA and cannot replace detailed autonomic testing, polysomnography, psychiatric assessment, or formal neuropsychological evaluation. Consequently, the NMSQ findings in our pooled analyses (particularly for the MSA cohort) must be interpreted as exploratory measures of self-reported symptom burden rather than as validated quantitative assessments of non-motor severity. Finally, although vigilance was continuously monitored during the EEG recordings via behavioral observation and live trace inspection, the absence of formal sleep staging means that brief episodes of subtle drowsiness or microsleeps cannot be definitively ruled out, representing a residual confounding factor. Future studies should validate these findings in larger, longitudinal cohorts utilizing standardized acquisition platforms, individual MRI-based source modeling and detailed, objective evaluation metrics for motor and non-motor symptoms, which may help clarify the functional network mechanisms and unravel the clinical implications of alpha slowing in PD and MSA.

### 4.4 Conclusion

In conclusion, this study demonstrates that resting-state cortical alpha slowing is present in PD and MSA-C, mainly detected in visual and dorsal attention networks. Chronological aging independently contributes to IAF slowing and amplifies the association between alpha slowing and non-motor symptom burden, particularly autonomic and psychiatric/cognitive symptoms. These findings support source-level IAF as a promising non-invasive marker of cortical network dysfunction in synucleinopathies, while emphasizing the importance of age-aware interpretation and MSA subtype stratification.

## Data Availability

The datasets presented in this study can be found in online repositories. The names of the repository/repositories and accession number(s) can be found at: https://github.com/chendaichao/individual-alpha-frequency-in-PD-and-MSA.

## References

[ref1] Braak H. Tredici K. D. Rüb U. de Vos R. A. I. Jansen Steur E. N. H. Braak E. (2003). Staging of brain pathology related to sporadic Parkinson’s disease. Neurobiol. Aging 24, 197–211. doi: 10.1016/S0197-4580(02)00065-9, 12498954

[ref2] Brissenden J. A. Tobyne S. M. Osher D. E. Levin E. J. Halko M. A. Somers D. C. (2018). Topographic Cortico-cerebellar networks revealed by visual attention and working memory. Curr. Biol. 28, 3364–3372.e5. doi: 10.1016/j.cub.2018.08.059, 30344119 PMC6257946

[ref3] Buckner R. L. Krienen F. M. Castellanos A. Diaz J. C. Yeo B. T. T. (2011). The organization of the human cerebellum estimated by intrinsic functional connectivity. J. Neurophysiol. 106, 2322–2345. doi: 10.1152/jn.00339.2011, 21795627 PMC3214121

[ref4] Cecchetti G. Agosta F. Canu E. Basaia S. Rugarli G. Curti D. G. . (2024). Analysis of individual alpha frequency in a large cohort from a tertiary memory center. Eur. J. Neurol. 31:e16424. doi: 10.1111/ene.16424, 39087560 PMC11414804

[ref5] Chen Z. Li G. Liu J. (2020). Autonomic dysfunction in Parkinson’s disease: implications for pathophysiology, diagnosis, and treatment. Neurobiol. Dis. 134:104700. doi: 10.1016/j.nbd.2019.104700, 31809788

[ref6] Chen Y. Wiesel A. Eldar Y. C. Hero A. O. (2010). Shrinkage algorithms for MMSE covariance estimation. IEEE Trans. Signal Process. 58, 5016–5029. doi: 10.1109/TSP.2010.2053029

[ref7] Dauwan M. Hoff J. I. Vriens E. M. Hillebrand A. Stam C. J. Sommer I. E. (2019). Aberrant resting-state oscillatory brain activity in Parkinson’s disease patients with visual hallucinations: an MEG source-space study. Neuroimage Clin 22:101752. doi: 10.1016/j.nicl.2019.101752, 30897434 PMC6425119

[ref8] de Cheveigné A. (2020). ZapLine: a simple and effective method to remove power line artifacts. NeuroImage 207:116356. doi: 10.1016/j.neuroimage.2019.116356, 31786167

[ref9] Deering R. Kaiser J. F. (2005). The use of a masking signal to improve empirical mode decomposition, in Proceedings. (ICASSP ‘05). IEEE International Conference on Acoustics, Speech, and Signal Processing, iv/485–iv/488, 4.

[ref10] Eichelberger D. Calabrese P. Meyer A. Chaturvedi M. Hatz F. Fuhr P. . (2017). Correlation of visuospatial ability and EEG slowing in patients with Parkinson’s disease. Parkinsons Dis 2017:3659784. doi: 10.1155/2017/3659784, 28348918 PMC5350347

[ref11] Fischl B. Sereno M. I. Tootell R. B. H. Dale A. M. (1999). High-resolution intersubject averaging and a coordinate system for the cortical surface. Hum. Brain Mapp. 8, 272–284. doi: 10.1002/(SICI)1097-0193(1999)8:4<272::AID-HBM10>3.0.CO;2-4, 10619420 PMC6873338

[ref12] Geraedts V. J. Boon L. I. Marinus J. Gouw A. A. van Hilten J. J. Stam C. J. . (2018). Clinical correlates of quantitative EEG in Parkinson disease: a systematic review. Neurology 91, 871–883. doi: 10.1212/WNL.0000000000006473, 30291182

[ref13] Gramfort A. Luessi M. Larson E. Engemann D. A. Strohmeier D. Brodbeck C. . (2013). MEG and EEG data analysis with MNE-python. Front. Neurosci. 7:267. doi: 10.3389/fnins.2013.00267, 24431986 PMC3872725

[ref14] Gray M. J. Emmanouil T. A. (2020). Individual alpha frequency increases during a task but is unchanged by alpha-band flicker. Psychophysiology 57:e13480. doi: 10.1111/psyp.13480, 31573080

[ref15] Haegens S. Cousijn H. Wallis G. Harrison P. J. Nobre A. C. (2014). Inter- and intra-individual variability in alpha peak frequency. NeuroImage 92, 46–55. doi: 10.1016/j.neuroimage.2014.01.049, 24508648 PMC4013551

[ref16] Halgren M. Ulbert I. Bastuji H. Fabó D. Erőss L. Rey M. . (2019). The generation and propagation of the human alpha rhythm. Proc. Natl. Acad. Sci. 116, 23772–23782. doi: 10.1073/pnas.1913092116, 31685634 PMC6876194

[ref17] Huang N. E. Shen Z. Long S. R. Wu M. C. Shih H. H. Zheng Q. . (1998). The empirical mode decomposition and the Hilbert spectrum for nonlinear and non-stationary time series analysis. Proc. A 454, 903–995. doi: 10.1098/rspa.1998.0193, 34341189

[ref18] Jaramillo-Jimenez A. Tovar-Rios D. A. Ospina J. A. Mantilla-Ramos Y.-J. Loaiza-López D. Henao Isaza V. . (2023). Spectral features of resting-state EEG in Parkinson’s disease: a multicenter study using functional data analysis. Clin. Neurophysiol. 151, 28–40. doi: 10.1016/j.clinph.2023.03.363, 37146531

[ref19] Kalia L. V. Lang A. E. (2015). Parkinson’s disease. Lancet 386, 896–912. doi: 10.1016/S0140-6736(14)61393-3, 25904081

[ref20] Katzman R. Zhang M. Ouang-Ya-Qu Wang Z. Liu W. T. Yu E. . (1988). A Chinese version of the mini-mental state examination; impact of illiteracy in a Shanghai dementia survey. J. Clin. Epidemiol. 41, 971–978. doi: 10.1016/0895-4356(88)90034-0, 3193141

[ref21] Klimesch W. Schimke H. Pfurtscheller G. (1993). Alpha frequency, cognitive load and memory performance. Brain Topogr. 5, 241–251. doi: 10.1007/BF01128991, 8507550

[ref22] Krismer F. Fanciulli A. Meissner W. G. Coon E. A. Wenning G. K. (2024). Multiple system atrophy: advances in pathophysiology, diagnosis, and treatment. Lancet Neurol. 23, 1252–1266. doi: 10.1016/S1474-4422(24)00396-X, 39577925

[ref23] Li A. Feitelberg J. Saini A. P. Höchenberger R. Scheltienne M. (2022). MNE-ICALabel: automatically annotating ICA components with ICLabel in python. J. Open Source Software 7:4484. doi: 10.21105/joss.04484

[ref24] Lin J. Xu X. Hou Y. Yang J. Shang H. (2020). Voxel-based meta-analysis of Gray matter abnormalities in multiple system atrophy. Front. Aging Neurosci. 12:591666. doi: 10.3389/fnagi.2020.591666, 33328969 PMC7729009

[ref25] Liu Z. de Zwart J. A. Yao B. van Gelderen P. Kuo L.-W. Duyn J. H. (2012). Finding thalamic BOLD correlates to posterior alpha EEG. NeuroImage 63, 1060–1069. doi: 10.1016/j.neuroimage.2012.08.025, 22986355 PMC3472152

[ref26] Liu H. Huang Z. Deng B. Chang Z. Yang X. Guo X. . (2023). QEEG signatures are associated with nonmotor dysfunctions in Parkinson’s disease and atypical parkinsonism: an integrative analysis. Aging Dis. 14, 204–218. doi: 10.14336/AD.2022.0514, 36818554 PMC9937709

[ref27] Luo Y. Yang N. Yang W. Chen B. Zhu S. Wu Y. . (2025). Autonomic dysfunction in multiple system atrophy: from pathophysiology to clinical manifestations. Ann. Med. 57:2488111. doi: 10.1080/07853890.2025.2488111, 40719373 PMC11983539

[ref28] Mantini D. Perrucci M. G. Del Gratta C. Romani G. L. Corbetta M. (2007). Electrophysiological signatures of resting state networks in the human brain. Proc. Natl. Acad. Sci. 104, 13170–13175. doi: 10.1073/pnas.0700668104, 17670949 PMC1941820

[ref29] Morita A. Kamei S. Mizutani T. (2011). Relationship between slowing of the EEG and cognitive impairment in Parkinson disease. J. Clin. Neurophysiol. 28, 384–387. doi: 10.1097/WNP.0b013e3182273211, 21811128

[ref30] Opie G. M. Hughes J. M. Puri R. (2024). Age-related differences in how the shape of alpha and beta oscillations change during reaction time tasks. Neurobiol. Aging 142, 52–64. doi: 10.1016/j.neurobiolaging.2024.08.001, 39153461

[ref31] Park J. Ho R. L. M. Wang W. Nguyen V. Q. Coombes S. A. (2024). The effect of age on alpha rhythms in the human brain derived from source localized resting-state electroencephalography. NeuroImage 292:120614. doi: 10.1016/j.neuroimage.2024.120614, 38631618

[ref32] Pascual-Marqui R. D. Lehmann D. Koukkou M. Kochi K. Anderer P. Saletu B. . (2011). Assessing interactions in the brain with exact low-resolution electromagnetic tomography. Philos. Trans. R. Soc. A Math. Phys. Eng. Sci. 369, 3768–3784. doi: 10.1098/rsta.2011.0081, 21893527

[ref33] Pascucci D. Menétrey M. Q. Passarotto E. Luo J. Paramento M. Rubega M. (2025). EEG brain waves and alpha rhythms: past, current and future direction. Neurosci. Biobehav. Rev. 176:106288. doi: 10.1016/j.neubiorev.2025.106288, 40669525

[ref34] Poewe W. Seppi K. Tanner C. M. Halliday G. M. Brundin P. Volkmann J. . (2017). Parkinson disease. Nat. Rev. Dis. Primers 3:17013. doi: 10.1038/nrdp.2017.13 28332488

[ref35] Poewe W. Stankovic I. Halliday G. Meissner W. G. Wenning G. K. Pellecchia M. T. . (2022). Multiple system atrophy. Nat. Rev. Dis. Primers 8:56. doi: 10.1038/s41572-022-00382-6, 36008429

[ref36] Postuma R. B. Berg D. Stern M. Poewe W. Olanow C. W. Oertel W. . (2015). MDS clinical diagnostic criteria for Parkinson’s disease. Mov. Disord. 30, 1591–1601. doi: 10.1002/mds.26424, 26474316

[ref37] Ren S. Zhang H. Zheng W. Liu M. Gao F. Wang Z. . (2018). Altered functional connectivity of Cerebello-cortical circuit in multiple system atrophy (cerebellar-type). Front. Neurosci. 12:996. doi: 10.3389/fnins.2018.00996, 30662394 PMC6328464

[ref38] Samaha J. Postle B. R. (2015). The speed of alpha-band oscillations predicts the temporal resolution of visual perception. Curr. Biol. 25, 2985–2990. doi: 10.1016/j.cub.2015.10.007, 26526370 PMC4654641

[ref39] Schaefer A. Kong R. Gordon E. M. Laumann T. O. Zuo X.-N. Holmes A. J. . (2018). Local-global Parcellation of the human cerebral cortex from intrinsic functional connectivity MRI. Cereb. Cortex 28, 3095–3114. doi: 10.1093/cercor/bhx179, 28981612 PMC6095216

[ref40] Schopp S. de Zeeuw J. Stotz S. Haberecht M. Weihrich K. S. Plotkin M. . (2025). EEG alpha peak frequency: cognitive impairment severity marker in isolated REM sleep behavior disorder. npj Parkinsons Dis. 11:185. doi: 10.1038/s41531-025-01059-z, 40593883 PMC12218050

[ref41] Seabold S. Perktold J. (2010). Statsmodels: econometric and statistical modeling with python, in 9th Python in Science Conference (SciPy).

[ref42] Soikkeli R. Partanen J. Soininen H. Pääkkönen A. Riekkinen P. (1991). Slowing of EEG in Parkinson’s disease. Electroencephalogr. Clin. Neurophysiol. 79, 159–165. doi: 10.1016/0013-4694(91)90134-p, 1714807

[ref43] Stern Y. (2012). Cognitive reserve in ageing and Alzheimer’s disease. Lancet Neurol. 11, 1006–1012. doi: 10.1016/S1474-4422(12)70191-6, 23079557 PMC3507991

[ref44] Stern Y. Arenaza-Urquijo E. M. Bartrés-Faz D. Belleville S. Cantilon M. Chetelat G. . (2020). Whitepaper: defining and investigating cognitive reserve, brain reserve, and brain maintenance. Alzheimers Dement. 16, 1305–1311. doi: 10.1016/j.jalz.2018.07.219, 30222945 PMC6417987

[ref45] Stoffers D. Bosboom J. L. W. Deijen J. B. Wolters E. C. Berendse H. W. Stam C. J. (2007). Slowing of oscillatory brain activity is a stable characteristic of Parkinson’s disease without dementia. Brain 130, 1847–1860. doi: 10.1093/brain/awm034, 17412733

[ref46] Surwillo W. W. (1963). The relation of simple response time to brain-wave frequency and the effects of age. Electroencephalogr. Clin. Neurophysiol. 15, 105–114. doi: 10.1016/0013-4694(63)90043-9, 13979350

[ref47] Tarasi L. Romei V. (2024). Individual alpha frequency contributes to the precision of human visual processing. J. Cogn. Neurosci. 36, 602–613. doi: 10.1162/jocn_a_02026, 37382485

[ref48] Turner C. Baylan S. Bracco M. Cruz G. Hanzal S. Keime M. . (2023). Developmental changes in individual alpha frequency: recording EEG data during public engagement events. *Imaging*. Neuroscience 1, 1–14. doi: 10.1162/imag_a_00001, 37719836 PMC10503479

[ref49] Virtanen P. Gommers R. Oliphant T. E. Haberland M. Reddy T. Cournapeau D. . (2020). SciPy 1.0: fundamental algorithms for scientific computing in python. Nat. Methods 17, 261–272. doi: 10.1038/s41592-019-0686-2, 32015543 PMC7056644

[ref50] Wang C. Hu J. Li P. Zhang M. Zhou L. Luo N. . (2024). Network disruption based on multi-modal EEG-MRI in α-synucleinopathies. Front. Neurol. 15:1442851. doi: 10.3389/fneur.2024.1442851, 39239399 PMC11374649

[ref51] Wenning G. K. Stankovic I. Vignatelli L. Fanciulli A. Calandra-Buonaura G. Seppi K. . (2022). The Movement Disorder Society criteria for the diagnosis of multiple system atrophy. Mov. Disord. 37, 1131–1148. doi: 10.1002/mds.29005, 35445419 PMC9321158

[ref52] Zhang Y. Lu Y. Wang D. Zhou C. Xu C. (2021). Relationship between individual alpha peak frequency and attentional performance in a multiple object tracking task among ice-hockey players. PLoS One 16:e0251443. doi: 10.1371/journal.pone.0251443, 34043652 PMC8158945

[ref53] Zhao Y. Cai J. Song J. Shi H. Kong W. Li X. . (2025a). Peak alpha frequency and alpha power spectral density as vulnerability markers of cognitive impairment in Parkinson’s disease: an exploratory EEG study. Front. Neurosci. 19:1575815. doi: 10.3389/fnins.2025.1575815, 40364859 PMC12069304

[ref54] Zhao Y. Shi H. Kong W. Wang X. Wei W. Zhan Z. . (2025b). Peak alpha frequency as an objective biomarker for cognitive assessment in post-stroke cognitive impairment. Front. Aging Neurosci. 17:1639970. doi: 10.3389/fnagi.2025.1639970, 41210901 PMC12589030

