# Supplementary material for: Resting-state cortical alpha frequency in Parkinson’s disease and multiple system atrophy: associations with diagnosis, age, and symptom burden
Source: Front Aging Neurosci. 2026 Sep 30;18:1928414. doi: 10.3389/fnagi.2026.1928414 (PMC13661991; doi:10.3389/fnagi.2026.1928414)
Supplement: Supplementary file 1 [file Data_Sheet_1.DOCX]

Supplementary Material

# 1 Supplementary Tables

**Supplementary Table S1. General linear model evaluating the main effects of clinical diagnosis and age on cortical mean IAF.**

**(A) Omnibus Main Effects.**

|  | ***df* (effect, error)** | ***F*** | ***P*** | **Partial *η*²** |
| --- | --- | --- | --- | --- |
| **Group** | 3, 95 | 3.594 | *0.016 | 0.102 |
| **Age** | 1, 95 | 8.026 | *0.006 | 0.078 |
| **Gender** | 1, 95 | 0.016 | 0.899 | 0.000 |
| **Device** | 1, 95 | 5.877 | *0.017 | 0.058 |

**(B) Model Coefficients.**

|  | ***β*** | **Standard Error** | **95% Confidence Interval** | ***t*(95)** | ***P*** |
| --- | --- | --- | --- | --- | --- |
| **Intercept**  (Grand Mean) | 9.937 | 0.191 | [9.559, 10.315] | 52.150 | <0.001 |
| **Group: PD** (deviation from grand mean) | -0.071 | 0.038 | [-0.145, 0.004] | -1.882 | 0.063 |
| **Group: MSA-P**  (deviation from grand mean) | 0.055 | 0.044 | [-0.031, 0.142] | 1.271 | 0.207 |
| **Group: MSA-C**  (deviation from grand mean) | -0.070 | 0.041 | [-0.152, 0.012] | -1.702 | 0.092 |
| **Age** (per year) | -0.008 | 0.003 | [-0.014, -0.002] | -2.833 | 0.006 |
| **Gender: Male**  (deviation from grand mean) | -0.003 | 0.023 | [-0.050, 0.044] | -0.127 | 0.899 |
| **Device: Neurofax EEG-1200**  (deviation from grand mean) | 0.055 | 0.023 | [0.010, 0.100] | 2.424 | 0.017 |

Results are derived from a general linear model (GLM) applied to the entire cohort (*n* = 102; comprising HC and patients with PD, MSA-P, and MSA-C). The model evaluated the main effects of group and age on the mean cortical IAF, while adjusting for gender and recording device. Categorical variables (group, gender, and recording device) were evaluated using sum coding (deviation coding). Under this coding scheme, diagnostic group had four levels and was represented by three contrast columns; gender and recording device were each represented by one contrast column. The intercept represents the adjusted grand mean IAF. The unstandardized coefficient (*β*) for a categorical level represents its adjusted deviation in Hz from the grand mean. The coefficient for the omitted category (e.g., “HC” for group, “Female” for gender) is not displayed but is mathematically equivalent to the negative sum of the coefficients of the other categories within that variable. For the continuous variable (age), *β* represents the adjusted change in IAF (in Hz) per one-year increase in age. Type III sums of squares were used for omnibus tests. Abbreviations: *df*, degrees of freedom. *, *P* < 0.05.

**Supplementary Table S2. Pairwise comparisons of cortical mean IAF among diagnostic groups.**

| **Contrast** | **Adjusted mean difference** | **Standard Error** | **95% CI** | ***t* (95)** | ***P* (uncorrected)** | ***P*  (FDR corrected)** | **Cohen’s *d*** |
| --- | --- | --- | --- | --- | --- | --- | --- |
| **PD − HC** | -0.156 | 0.058 | [-0.271, -0.041] | -2.692 | 0.008 | *0.044 | -0.700 |
| **MSA-P − HC** | -0.030 | 0.066 | [-0.161, 0.102] | -0.452 | 0.652 | 0.783 | -0.135 |
| **MSA-C − HC** | -0.156 | 0.063 | [-0.280, -0.031] | -2.486 | 0.015 | *0.044 | -0.698 |
| **MSA-P − PD** | 0.126 | 0.067 | [-0.007, 0.259] | 1.885 | 0.062 | 0.122 | 0.566 |
| **MSA-C − PD** | 0.001 | 0.064 | [-0.127, 0.128] | 0.008 | 0.994 | 0.994 | 0.002 |
| **MSA-C − MSA-P** | -0.126 | 0.071 | [-0.267, 0.016] | -1.761 | 0.081 | 0.122 | -0.564 |

Contrasts were estimated from the global IAF general linear model (GLM) adjusted for age, sex, and recording device. Negative values indicate lower IAF in the first-listed group. *P* values were corrected across all six pairwise group contrasts using the False Discovery Rate (FDR) procedure. All pairwise contrasts were evaluated using the residual degrees of freedom from the GLM (*df* = 95). *, *P* < 0.05.

**Supplementary Table S3. Analysis of effect of recording device on IAF.**

**(A) Assessment of the group × device interaction effect on cortical mean IAF.**

|  | ***df* (effect, error)** | ***F*** | ***P*** | **Partial *η*²** |
| --- | --- | --- | --- | --- |
| **Group** | 3, 92 | 3.244 | 0.026 | 0.096 |
| **Age** | 1, 92 | 8.912 | 0.004 | 0.088 |
| **Gender** | 1, 92 | 0.013 | 0.909 | 0.000 |
| **Device** | 1, 92 | 4.896 | 0.029 | 0.051 |
| **Group × Device** | 3, 92 | 0.590 | 0.623 | 0.019 |

Results are derived from a supplementary general linear model (GLM) applied to the entire cohort (*n* = 102). This model included the group × device interaction term to determine whether the diagnostic effects differed by recording system.

**(B) Device-stratified pairwise comparisons of cortical mean IAF among groups.**

|  | **Contrast** | **Adjusted mean difference** | **Standard Error** | **95%  Confidence Interval** | ***t*** | ***P* (uncorrected)** | ***P*  (FDR corrected)** | **Cohen's *d*** |
| --- | --- | --- | --- | --- | --- | --- | --- | --- |
| **Device 1: Neurofax EEG-1200 (Nihon Kohden)** | PD − HC | -0.178 | 0.073 | [-0.324, -0.032] | -2.443 | 0.018 | 0.109 | -0.832 |
|  | MSA-P − HC | -0.110 | 0.087 | [-0.284, 0.064] | -1.267 | 0.211 | 0.422 | -0.514 |
|  | MSA-C − HC | -0.152 | 0.088 | [-0.328, 0.024] | -1.731 | 0.090 | 0.269 | -0.710 |
|  | MSA-P − PD | 0.068 | 0.087 | [-0.107, 0.243] | 0.779 | 0.440 | 0.660 | 0.318 |
|  | MSA-C − PD | 0.026 | 0.089 | [-0.153, 0.206] | 0.293 | 0.771 | 0.771 | 0.122 |
|  | MSA-C − MSA-P | -0.042 | 0.100 | [-0.242, 0.158] | -0.420 | 0.676 | 0.771 | -0.196 |
| **Device 2:  PN-NET (Yunshen)** | PD − HC | -0.111 | 0.102 | [-0.316, 0.094] | -1.092 | 0.281 | 0.422 | -0.466 |
|  | MSA-P − HC | 0.072 | 0.111 | [-0.153, 0.296] | 0.644 | 0.523 | 0.628 | 0.300 |
|  | MSA-C − HC | -0.145 | 0.094 | [-0.335, 0.044] | -1.550 | 0.129 | 0.258 | -0.610 |
|  | MSA-P − PD | 0.183 | 0.106 | [-0.031, 0.396] | 1.725 | 0.092 | 0.258 | 0.766 |
|  | MSA-C − PD | -0.034 | 0.099 | [-0.235, 0.166] | -0.347 | 0.731 | 0.731 | -0.144 |
|  | MSA-C − MSA-P | -0.217 | 0.109 | [-0.437, 0.003] | -1.992 | 0.053 | 0.258 | -0.910 |

Contrasts were estimated from the global IAF general linear model (GLM) adjusted for age and sex, applied separately for the cohort of the two recording devices (Device 1: *n* = 55; Device 2: *n* = 47). *P* values were corrected across all six pairwise group contrasts using FDR procedure within the cohort of each recording device. All pairwise contrasts were evaluated using the residual degrees of freedom from the GLM (*df* = 49 for Device 1 and 41 for Device 2).

**Supplementary Table S4. Main effects of clinical diagnostic group on network-level IAF.**

| **Network** | ***df***  **(effect, error)** | **Group *F*** | ***P* value  (raw)** | ***P* value**  **(FDR-corrected)** | **Partial *η*²** |
| --- | --- | --- | --- | --- | --- |
| VN | 3, 95 | 3.967 | 0.010 | *0.036 | 0.111 |
| DAN | 3, 95 | 4.080 | 0.009 | *0.036 | 0.114 |
| SMN | 3, 95 | 3.140 | 0.029 | *0.044 | 0.090 |
| SVAN | 3, 95 | 3.007 | 0.034 | *0.044 | 0.087 |
| FPN | 3, 95 | 2.875 | 0.040 | *0.044 | 0.083 |
| DMN | 3, 95 | 3.183 | 0.027 | *0.044 | 0.091 |
| LN | 3, 95 | 2.802 | 0.044 | *0.044 | 0.081 |

Network-level IAF was analyzed separately for each of the seven canonical cortical networks using a general linear model (GLM) including diagnostic group, age, sex, and recording device as predictors, applied to the entire cohort (*n* = 102). The reported *F* statistics and partial *η*² values correspond to the omnibus main effect of diagnostic group. Raw *P* values were corrected across the seven network-level group tests using the FDR procedure. *, *P* < 0.05.

**Supplementary Table S5. Pairwise comparisons of network-level IAF among diagnostic groups.**

|  | **Contrast** | **Adjusted mean difference** | **Standard Error** | **95%  Confidence Interval** | ***t*(95)** | ***P* (uncorrected)** | ***P*  (FDR-corrected)** | **Cohen's *d*** |
| --- | --- | --- | --- | --- | --- | --- | --- | --- |
| **VN** | PD − HC | -0.190 | 0.070 | [-0.328, -0.052] | -2.736 | 0.007 | *0.031 | -0.712 |
|  | MSA-P − HC | -0.026 | 0.079 | [-0.184, 0.131] | -0.330 | 0.742 | 0.891 | -0.098 |
|  | MSA-C − HC | -0.196 | 0.075 | [-0.345, -0.048] | -2.619 | 0.010 | *0.031 | -0.735 |
|  | MSA-P − PD | 0.164 | 0.080 | [0.005, 0.323] | 2.045 | 0.044 | 0.074 | 0.614 |
|  | MSA-C − PD | -0.006 | 0.077 | [-0.159, 0.147] | -0.081 | 0.936 | 0.936 | -0.023 |
|  | MSA-C − MSA-P | -0.170 | 0.085 | [-0.340, -0.001] | -1.992 | 0.049 | 0.074 | -0.637 |
| **DAN** | PD − HC | -0.192 | 0.064 | [-0.319, -0.065] | -3.011 | 0.003 | *0.020 | -0.784 |
|  | MSA-P − HC | -0.036 | 0.073 | [-0.181, 0.109] | -0.492 | 0.624 | 0.745 | -0.146 |
|  | MSA-C − HC | -0.169 | 0.069 | [-0.306, -0.032] | -2.455 | 0.016 | *0.048 | -0.690 |
|  | MSA-P − PD | 0.156 | 0.074 | [0.010, 0.302] | 2.123 | 0.036 | 0.073 | 0.637 |
|  | MSA-C − PD | 0.023 | 0.071 | [-0.118, 0.164] | 0.326 | 0.745 | 0.745 | 0.094 |
|  | MSA-C − MSA-P | -0.133 | 0.078 | [-0.289, 0.023] | -1.698 | 0.093 | 0.139 | -0.543 |
| **SMN** | PD − HC | -0.167 | 0.064 | [-0.294, -0.040] | -2.604 | 0.011 | 0.064 | -0.677 |
|  | MSA-P − HC | -0.044 | 0.073 | [-0.189, 0.102] | -0.596 | 0.553 | 0.663 | -0.177 |
|  | MSA-C − HC | -0.160 | 0.069 | [-0.298, -0.023] | -2.325 | 0.022 | 0.067 | -0.653 |
|  | MSA-P − PD | 0.123 | 0.074 | [-0.024, 0.269] | 1.667 | 0.099 | 0.198 | 0.500 |
|  | MSA-C − PD | 0.006 | 0.071 | [-0.135, 0.147] | 0.085 | 0.932 | 0.932 | 0.025 |
|  | MSA-C − MSA-P | -0.117 | 0.079 | [-0.273, 0.039] | -1.486 | 0.141 | 0.211 | -0.476 |
| **SVAN** | PD − HC | -0.142 | 0.057 | [-0.255, -0.028] | -2.472 | 0.015 | 0.081 | -0.643 |
|  | MSA-P − HC | -0.026 | 0.065 | [-0.155, 0.104] | -0.390 | 0.697 | 0.837 | -0.116 |
|  | MSA-C − HC | -0.139 | 0.062 | [-0.261, -0.016] | -2.246 | 0.027 | 0.081 | -0.631 |
|  | MSA-P − PD | 0.116 | 0.066 | [-0.015, 0.247] | 1.756 | 0.082 | 0.165 | 0.527 |
|  | MSA-C − PD | 0.003 | 0.064 | [-0.123, 0.129] | 0.043 | 0.965 | 0.965 | 0.013 |
|  | MSA-C − MSA-P | -0.113 | 0.070 | [-0.253, 0.027] | -1.608 | 0.111 | 0.167 | -0.515 |
| **FPN** | PD − HC | -0.134 | 0.054 | [-0.242, -0.026] | -2.465 | 0.015 | 0.093 | -0.641 |
|  | MSA-P − HC | -0.023 | 0.062 | [-0.146, 0.100] | -0.369 | 0.713 | 0.855 | -0.110 |
|  | MSA-C − HC | -0.125 | 0.059 | [-0.241, -0.008] | -2.122 | 0.036 | 0.109 | -0.596 |
|  | MSA-P − PD | 0.111 | 0.063 | [-0.013, 0.236] | 1.771 | 0.080 | 0.160 | 0.532 |
|  | MSA-C − PD | 0.009 | 0.060 | [-0.110, 0.129] | 0.157 | 0.875 | 0.875 | 0.045 |
|  | MSA-C − MSA-P | -0.102 | 0.067 | [-0.234, 0.031] | -1.519 | 0.132 | 0.198 | -0.486 |
| **DMN** | PD − HC | -0.129 | 0.053 | [-0.235, -0.024] | -2.434 | 0.017 | 0.054 | -0.633 |
|  | MSA-P − HC | -0.022 | 0.061 | [-0.142, 0.099] | -0.361 | 0.719 | 0.862 | -0.107 |
|  | MSA-C − HC | -0.138 | 0.057 | [-0.252, -0.024] | -2.410 | 0.018 | 0.054 | -0.677 |
|  | MSA-P − PD | 0.107 | 0.061 | [-0.014, 0.229] | 1.752 | 0.083 | 0.124 | 0.526 |
|  | MSA-C − PD | -0.009 | 0.059 | [-0.126, 0.108] | -0.150 | 0.881 | 0.881 | -0.043 |
|  | MSA-C − MSA-P | -0.116 | 0.065 | [-0.246, 0.013] | -1.779 | 0.078 | 0.124 | -0.569 |
| **LN** | PD − HC | -0.122 | 0.058 | [-0.237, -0.006] | -2.093 | 0.039 | 0.117 | -0.545 |
|  | MSA-P − HC | -0.054 | 0.067 | [-0.186, 0.078] | -0.816 | 0.416 | 0.500 | -0.243 |
|  | MSA-C − HC | -0.166 | 0.063 | [-0.290, -0.041] | -2.635 | 0.010 | 0.059 | -0.740 |
|  | MSA-P − PD | 0.068 | 0.067 | [-0.066, 0.201] | 1.006 | 0.317 | 0.476 | 0.302 |
|  | MSA-C − PD | -0.044 | 0.065 | [-0.172, 0.085] | -0.676 | 0.501 | 0.501 | -0.195 |
|  | MSA-C − MSA-P | -0.111 | 0.072 | [-0.253, 0.031] | -1.554 | 0.124 | 0.247 | -0.497 |

Pairwise group contrasts were estimated separately for each of the seven canonical cortical networks using the general linear model (GLM) including diagnostic group, age, gender, and recording device as predictors, applied to the entire cohort (*n* = 102). The adjusted mean difference represents the estimated IAF difference between the first and second groups listed in each contrast. Negative values indicate lower IAF in the first-listed group. All pairwise contrasts were evaluated using the residual degrees of freedom from the GLM (*df* = 95). Within each of the seven networks, raw *P* values were corrected across the six group contrasts using the FDR procedure. *, *P* < 0.05.

**Supplementary Table S6. Linear mixed-effects model testing whether network-level cortical IAF profiles differed across diagnostic groups.**

|  | **𝜒²** | ***df*** | ***P* value** |
| --- | --- | --- | --- |
| **Group** | 10.522 | 3 | *0.015 |
| **Network** | 111.412 | 6 | <0.001 |
| **Group × Network** | 27.255 | 18 | 0.074 |
| **Age** | 8.170 | 1 | *0.004 |
| **Gender** | 0.031 | 1 | 0.861 |
| **Device** | 6.464 | 1 | *0.011 |

Network-level IAF was modeled as a repeated within-participant outcome. The model included group, network, group × network interaction, age, gender, and recording device as fixed effects, with a participant-specific random intercept. Fixed effects were evaluated using joint Wald Chi-squared tests. *, *P* < 0.05.

**Supplementary Table S7. Assessment of the group × age interaction effect on cortical mean IAF.**

|  | ***df* (effect, error)** | ***F*** | ***P*** | **Partial *η*²** |
| --- | --- | --- | --- | --- |
| **Group** | 3, 92 | 4.108 | *0.009 | 0.118 |
| **Age** | 1, 92 | 8.980 | *0.004 | 0.089 |
| **Group × Age** | 3, 92 | 1.564 | 0.203 | 0.049 |
| **Gender** | 1, 92 | 0.003 | 0.959 | 0.000 |
| **Device** | 1, 92 | 4.911 | *0.029 | 0.051 |

Results are derived from a supplementary general linear model (GLM) applied to the entire cohort (*n* = 102). This model included the group × age interaction term to determine whether the rate of age-related alpha slowing differed among the clinical groups. *, *P* < 0.05.

**Supplementary Table S8. Tests of diagnostic heterogeneity to justify pooling the patient cohort.**

**(A) Assessment of group×age×symptom score interaction on cortical IAF, for examining diagnostic-group heterogeneity in the age-dependent associations between symptom burden and IAF.**

|  |  | ***df*  (effect, error)** | **Group×Age×Score *F*** | ***P*  (Uncorrected)** | ***P*  (FDR-corrected)** | **Partial *η*²** |
| --- | --- | --- | --- | --- | --- | --- |
| **Non-motor symptom** | **NMSQ total** | 2, 43 | 0.923 | 0.405 | 0.540 | 0.041 |
|  | **Autonomic** | 2, 43 | 0.432 | 0.652 | 0.652 | 0.020 |
|  | **Sleep** | 2, 43 | 2.148 | 0.129 | 0.258 | 0.091 |
|  | **Psychiatric & Cognitive** | 2, 43 | 2.227 | 0.120 | 0.258 | 0.094 |
| **Motor symptom** | **MDS-UPDRSIII total** | 2, 53 | 0.144 | 0.866 | 0.866 | 0.005 |
|  | **Bradykinesia** | 2, 53 | 0.676 | 0.513 | 0.866 | 0.025 |
|  | **Rigidity** | 2, 53 | 0.687 | 0.507 | 0.866 | 0.025 |
|  | **Midline** | 2, 53 | 0.273 | 0.762 | 0.866 | 0.010 |
|  | **Tremor** | 2, 53 | 0.516 | 0.600 | 0.866 | 0.019 |

For each subdomain score, global cortical IAF was analyzed using a full factorial general linear model (GLM) containing diagnostic group, age, symptom score, all corresponding two-way interactions, and the group × age × symptom interaction, with gender and recording device included as covariates. The analysis for non-motor and motor symptoms included patients with NMSQ data (*n* = 57) and patients with MDS-UPDRSIII data (*n* = 67). The reported statistics correspond to the omnibus group × age × symptom interaction. FDR correction was applied separately across the four NMSQ scores and across the five MDS-UPDRSIII scores.

**(B) Assessment of group×symptom score and group×age interaction on cortical IAF, for examining diagnostic-group heterogeneity in the symptom–IAF and age–IAF relationship.**

|  |  | **Group×Score** | | | | **Group×Age** | | | |
| --- | --- | --- | --- | --- | --- | --- | --- | --- | --- |
|  |  | ***df*  (effect, error)** | ***F*** | ***P***  **(Uncorrected)** | ***P*  (FDR-corrected)** | ***df*  (effect, error)** | ***F*** | ***P* (Uncorrected)** | ***P*  (FDR-corrected)** |
| **Non-motor symptom** | **NMSQ total** | 2, 45 | 1.443 | 0.247 | 0.494 | 2, 45 | 1.126 | 0.333 | 0.444 |
|  | **Autonomic** | 2, 45 | 1.508 | 0.232 | 0.494 | 2, 45 | 0.576 | 0.566 | 0.566 |
|  | **Sleep** | 2, 45 | 0.965 | 0.389 | 0.518 | 2, 45 | 1.619 | 0.210 | 0.419 |
|  | **Psychiatric & Cognitive** | 2, 45 | 0.005 | 0.995 | 0.995 | 2, 45 | 2.093 | 0.135 | 0.419 |
| **Motor symptom** | **MDS-UPDRSIII total** | 2, 55 | 1.217 | 0.304 | 0.582 | 2, 55 | 0.536 | 0.588 | 0.595 |
|  | **Bradykinesia** | 2, 55 | 4.578 | 0.014 | 0.072 | 2, 55 | 0.979 | 0.382 | 0.595 |
|  | **Rigidity** | 2, 55 | 0.349 | 0.707 | 0.707 | 2, 55 | 1.406 | 0.254 | 0.595 |
|  | **Midline** | 2, 55 | 1.072 | 0.349 | 0.582 | 2, 55 | 0.525 | 0.595 | 0.595 |
|  | **Tremor** | 2, 55 | 0.446 | 0.642 | 0.707 | 2, 55 | 1.482 | 0.236 | 0.595 |

For each subdomain score, global cortical IAF was analyzed using a general linear model (GLM) containing diagnostic group, age, symptom score, the group×symptom score, the group×age, and the age×symptom score interaction, with gender and recording device included as covariates. The reported statistics correspond to the omnibus group×symptom and group×age interaction. FDR correction was applied separately across the four NMSQ scores and across the five MDS-UPDRSIII scores.

**Supplementary Table S9. Age-dependent associations between clinical symptom burden and global cortical IAF.**

|  |  | ***β* (Age ×Score)** | **SE** | **95%  CI** | ***df*** | ***t*** | ***P***  **(Uncor- rected)** | ***P*  (FDR-corrected)** | **Partial  *η*²** |
| --- | --- | --- | --- | --- | --- | --- | --- | --- | --- |
| **Non-motor symptom** | **NMSQ total** | -0.0017 | 0.0005 | [-0.0026, -0.0008] | 49 | -3.658 | < 0.001 | **0.002 | 0.215 |
|  | **Autonomic** | -0.0034 | 0.0011 | [-0.0056, -0.0012] | 49 | -3.084 | 0.003 | **0.007 | 0.163 |
|  | **Sleep** | -0.0023 | 0.0014 | [-0.0050, 0.0005] | 49 | -1.673 | 0.101 | 0.101 | 0.054 |
|  | **Psychiatric  & Cognitive** | -0.0049 | 0.0020 | [-0.0089, -0.0009] | 49 | -2.486 | 0.016 | *0.022 | 0.112 |
| **Motor symptom** | **MDS-UPDRSIII total** | -0.0005 | 0.0002 | [-0.0010, -0.0001] | 59 | -2.390 | 0.020 | 0.051 | 0.088 |
|  | **Bradykinesia** | -0.0010 | 0.0005 | [-0.0019, -0.0001] | 59 | -2.244 | 0.029 | 0.051 | 0.079 |
|  | **Rigidity** | -0.0006 | 0.0009 | [-0.0024, 0.0013] | 59 | -0.618 | 0.539 | 0.539 | 0.006 |
|  | **Midline** | -0.0017 | 0.0008 | [-0.0033, -0.0002] | 59 | -2.217 | 0.030 | 0.051 | 0.077 |
|  | **Tremor** | -0.0015 | 0.0011 | [-0.0038, 0.0008] | 59 | -1.325 | 0.190 | 0.238 | 0.029 |

For each subdomain score, global cortical IAF was analyzed using a general linear model (GLM) containing diagnostic group, age, symptom score, and the age×symptom score interaction, with gender and recording device included as covariates. The analysis for non-motor and motor symptoms included patients with NMSQ data (*n* = 57) and patients with MDS-UPDRSIII data (*n* = 67). A negative *T* statistic indicates that advancing age worsens the negative correlation between symptom burden and IAF. FDR correction was applied separately across the four NMSQ scores and across the five MDS-UPDRSIII scores. Abbreviations: SE, standard error; CI, confidence interval; *df*, degrees of freedom. *, *P* < 0.05; **, *P* < 0.01.

**Supplementary Table S10. Age-dependent associations between non-motor symptom burden and cortical network-level IAF.**

| **Non-motor Symptom** | **Network** | ***β* (Age × Symptom)** | **SE** | **95% CI** | ***t*** | ***df*** | ***P*  (Uncor-rected)** | ***P*  (FDR-corrected)** | **Partial *η*²** |
| --- | --- | --- | --- | --- | --- | --- | --- | --- | --- |
| **NMSQ total** | **VN** | -0.0019 | 0.0006 | [-0.0030, -0.0007] | -3.258 | 49 | 0.002 | **0.006 | 0.178 |
|  | **DAN** | -0.0018 | 0.0005 | [-0.0028, -0.0008] | -3.524 | 49 | < 0.001 | **0.005 | 0.202 |
|  | **SMN** | -0.0018 | 0.0005 | [-0.0028, -0.0008] | -3.582 | 49 | < 0.001 | **0.005 | 0.208 |
|  | **SVAN** | -0.0015 | 0.0004 | [-0.0024, -0.0006] | -3.408 | 49 | 0.001 | **0.006 | 0.192 |
|  | **FPN** | -0.0017 | 0.0004 | [-0.0025, -0.0008] | -3.923 | 49 | < 0.001 | **0.005 | 0.239 |
|  | **DMN** | -0.0015 | 0.0004 | [-0.0023, -0.0007] | -3.609 | 49 | < 0.001 | **0.005 | 0.210 |
|  | **LN** | -0.0016 | 0.0004 | [-0.0024, -0.0007] | -3.527 | 49 | < 0.001 | **0.005 | 0.202 |
| **Autonomic** | **VN** | -0.0031 | 0.0014 | [-0.0059, -0.0003] | -2.250 | 49 | 0.029 | *0.046 | 0.094 |
|  | **DAN** | -0.0040 | 0.0012 | [-0.0064, -0.0015] | -3.258 | 49 | 0.002 | **0.006 | 0.178 |
|  | **SMN** | -0.0039 | 0.0012 | [-0.0064, -0.0015] | -3.222 | 49 | 0.002 | **0.006 | 0.175 |
|  | **SVAN** | -0.0035 | 0.0011 | [-0.0056, -0.0014] | -3.329 | 49 | 0.002 | **0.006 | 0.184 |
|  | **FPN** | -0.0034 | 0.0010 | [-0.0055, -0.0014] | -3.353 | 49 | 0.002 | **0.006 | 0.187 |
|  | **DMN** | -0.0030 | 0.0010 | [-0.0050, -0.0010] | -3.020 | 49 | 0.004 | **0.009 | 0.157 |
|  | **LN** | -0.0028 | 0.0011 | [-0.0049, -0.0007] | -2.635 | 49 | 0.011 | *0.022 | 0.124 |
| **Sleep** | **VN** | -0.0026 | 0.0016 | [-0.0059, 0.0007] | -1.583 | 49 | 0.120 | 0.131 | 0.049 |
|  | **DAN** | -0.0022 | 0.0015 | [-0.0053, 0.0008] | -1.462 | 49 | 0.150 | 0.156 | 0.042 |
|  | **SMN** | -0.0024 | 0.0015 | [-0.0054, 0.0007] | -1.573 | 49 | 0.122 | 0.131 | 0.048 |
|  | **SVAN** | -0.0019 | 0.0013 | [-0.0045, 0.0008] | -1.433 | 49 | 0.158 | 0.158 | 0.040 |
|  | **FPN** | -0.0023 | 0.0013 | [-0.0048, 0.0003] | -1.794 | 49 | 0.079 | 0.096 | 0.062 |
|  | **DMN** | -0.0022 | 0.0012 | [-0.0046, 0.0002] | -1.823 | 49 | 0.074 | 0.095 | 0.064 |
|  | **LN** | -0.0022 | 0.0013 | [-0.0048, 0.0004] | -1.707 | 49 | 0.094 | 0.110 | 0.056 |
| **Psychiatric & Cognitive** | **VN** | -0.0072 | 0.0023 | [-0.0118, -0.0026] | -3.159 | 49 | 0.003 | **0.006 | 0.169 |
|  | **DAN** | -0.0054 | 0.0022 | [-0.0098, -0.0010] | -2.465 | 49 | 0.017 | *0.032 | 0.110 |
|  | **SMN** | -0.0049 | 0.0022 | [-0.0094, -0.0005] | -2.218 | 49 | 0.031 | *0.046 | 0.091 |
|  | **SVAN** | -0.0040 | 0.0020 | [-0.0079, -0.0001] | -2.050 | 49 | 0.046 | 0.061 | 0.079 |
|  | **FPN** | -0.0045 | 0.0019 | [-0.0082, -0.0008] | -2.445 | 49 | 0.018 | *0.032 | 0.109 |
|  | **DMN** | -0.0037 | 0.0018 | [-0.0073, -0.0001] | -2.072 | 49 | 0.044 | 0.061 | 0.081 |
|  | **LN** | -0.0043 | 0.0019 | [-0.0081, -0.0004] | -2.229 | 49 | 0.030 | *0.046 | 0.092 |

The general linear model (GLM) for each network and each non-motor symptom domain included group, age, symptom score, the age × symptom score interaction, gender, and recording device as predictors. The 28 models (7 networks combined with 4 symptom subdomains) were corrected jointly using the FDR correction. *, *P* < 0.05; **, *P*< 0.01.

**Supplementary Table S11. Detailed information about the EEG recording system.**

|  | **Neurofax EEG-1200 (Nihon Kohden)** | **PN-NET (Yunshen)** |
| --- | --- | --- |
| **Connector** | JE213A (64 channels) | PN-NET (64 channels) |
| **Noise level** | ≤ 1.5 μVp-p | ≤ 1.1 μVp-p |
| **Common mode rejection ratio (CMMR)** | ≥ 110 dB (at 60Hz) | ≥ 110 dB |
| **Time constant (High‑pass filter)** | 2s (0.08 Hz) | 0.25s (0.64 Hz) |
| **High frequency (Bandwidth)** | 3000 Hz | 3000 Hz |
| **Offset tolerance** | ± 1000 mV | ± 300 mV |
| **Input range** | ± 3.2 mV | ± 16 mV |
| **AD conversion** | 16 bits (97nV/LSB) | 18 bits (122nV/LSB) |
| **Online reference** | AFz | Fpz |

**Supplementary Table S12. Detailed information about EEG quality control metrics for all participants.**

| **ID** | **Group** | **Age (y)** | **Gender** | **Disease duration (y)** | **Number of interpolated channels** | **Number of rejected ICA components** | **Number  of epochs** | **Duration of EEG covered by epochs (s)** |
| --- | --- | --- | --- | --- | --- | --- | --- | --- |
| 1 | PD | 72.3 | F | 3.0 | 5 | 4 | 38 | 156 |
| 2 | PD | 75.9 | M | 6.3 | 1 | 6 | 43 | 176 |
| 3 | PD | 70.2 | F | 3.8 | 5 | 1 | 38 | 160 |
| 4 | PD | 67.9 | M | 0.3 | 4 | 1 | 42 | 172 |
| 5 | PD | 71.8 | F | 7.4 | 2 | 12 | 39 | 164 |
| 6 | PD | 49.6 | F | 1.4 | 3 | 5 | 44 | 180 |
| 7 | MSA-C | 68.5 | M | 5.0 | 3 | 6 | 40 | 164 |
| 8 | MSA-P | 50.3 | F | 1.2 | 2 | 11 | 36 | 152 |
| 9 | MSA-C | 67.7 | M | 2.3 | 3 | 7 | 43 | 176 |
| 10 | MSA-P | 69.8 | M | 4.4 | 3 | 11 | 39 | 164 |
| 11 | MSA-C | 66.0 | M | 4.1 | 2 | 4 | 39 | 164 |
| 12 | MSA-P | 83.3 | M | 6.6 | 1 | 6 | 36 | 160 |
| 13 | PD | 61.0 | M | 1.8 | 1 | 5 | 38 | 168 |
| 14 | PD | 75.9 | M | 3.5 | 7 | 2 | 44 | 180 |
| 15 | PD | 78.2 | M | 1.5 | 6 | 5 | 42 | 172 |
| 16 | PD | 66.4 | M | 6.5 | 5 | 2 | 44 | 180 |
| 17 | PD | 65.5 | M | 4.1 | 2 | 9 | 44 | 180 |
| 18 | PD | 65.4 | F | 3.7 | 2 | 3 | 41 | 180 |
| 19 | MSA-P | 61.8 | F | 1.1 | 3 | 8 | 44 | 180 |
| 20 | MSA-C | 55.8 | M | 0.7 | 11 | 4 | 40 | 168 |
| 21 | MSA-C | 62.2 | F | 1.8 | 1 | 5 | 42 | 176 |
| 22 | MSA-P | 52.6 | F | 3.5 | 3 | 13 | 41 | 172 |
| 23 | MSA-C | 63.9 | F | 1.4 | 6 | 3 | 44 | 180 |
| 24 | MSA-P | 70.6 | M | 2.2 | 6 | 2 | 44 | 180 |
| 25 | MSA-C | 76.2 | F | 3.0 | 4 | 0 | 43 | 176 |
| 26 | PD | 69.6 | M | 7.8 | 4 | 2 | 44 | 180 |
| 27 | PD | 52.4 | M | 2.6 | 3 | 7 | 44 | 184 |
| 28 | MSA-P | 70.8 | F | 0.9 | 6 | 4 | 40 | 164 |
| 29 | MSA-P | 60.7 | M | 7.3 | 4 | 1 | 44 | 184 |
| 30 | MSA-C | 63.8 | M | 1.2 | 5 | 4 | 44 | 180 |
| 31 | MSA-C | 53.2 | M | 1.6 | 2 | 11 | 44 | 180 |
| 32 | PD | 49.4 | M | 7.9 | 5 | 7 | 43 | 176 |
| 33 | PD | 62.7 | F | 4.7 | 7 | 1 | 42 | 172 |
| 34 | MSA-P | 57.9 | F | 8.5 | 4 | 9 | 27 | 124 |
| 35 | MSA-P | 67.7 | F | 4.5 | 4 | 4 | 39 | 168 |
| 36 | HC | 72.5 | F | - | 1 | 4 | 37 | 152 |
| 37 | HC | 72.4 | F | - | 2 | 0 | 41 | 168 |
| 38 | HC | 75.9 | F | - | 6 | 2 | 43 | 180 |
| 39 | HC | 64.0 | M | - | 4 | 2 | 37 | 156 |
| 40 | HC | 77.2 | M | - | 7 | 12 | 34 | 152 |
| 41 | HC | 63.1 | M | - | 7 | 9 | 35 | 148 |
| 42 | HC | 63.2 | M | - | 3 | 8 | 42 | 180 |
| 43 | HC | 72.9 | M | - | 2 | 2 | 44 | 180 |
| 44 | HC | 56.7 | M | - | 5 | 2 | 43 | 176 |
| 45 | HC | 74.3 | M | - | 10 | 4 | 40 | 168 |
| 46 | HC | 65.2 | F | - | 9 | 5 | 41 | 172 |
| 47 | HC | 73.1 | M | - | 3 | 20 | 44 | 180 |
| 48 | HC | 72.2 | M | - | 5 | 6 | 37 | 160 |
| 49 | HC | 63.9 | M | - | 2 | 3 | 34 | 152 |
| 50 | HC | 73.2 | M | - | 1 | 1 | 44 | 180 |
| 51 | HC | 63.0 | M | - | 5 | 15 | 43 | 180 |
| 52 | HC | 76.2 | M | - | 0 | 6 | 43 | 180 |
| 53 | HC | 66.4 | M | - | 1 | 3 | 44 | 180 |
| 54 | HC | 60.8 | F | - | 0 | 4 | 44 | 180 |
| 55 | HC | 68.3 | M | - | 2 | 6 | 44 | 188 |
| 56 | MSA-C | 59.4 | F | 4.0 | 7 | 3 | 37 | 164 |
| 57 | PD | 73.0 | M | 8.0 | 2 | 24 | 44 | 180 |
| 58 | PD | 74.7 | M | 4.8 | 2 | 7 | 44 | 180 |
| 59 | MSA-C | 63.1 | M | 0.5 | 3 | 3 | 44 | 180 |
| 60 | MSA-C | 66.0 | M | 1.4 | 5 | 2 | 44 | 184 |
| 61 | PD | 69.3 | M | 9.9 | 3 | 3 | 44 | 180 |
| 62 | MSA-C | 55.4 | F | 1.1 | 2 | 7 | 44 | 184 |
| 63 | MSA-C | 76.1 | M | 2.4 | 6 | 2 | 44 | 192 |
| 64 | MSA-P | 68.1 | M | 2.1 | 9 | 6 | 44 | 180 |
| 65 | MSA-C | 45.8 | F | 0.3 | 6 | 4 | 44 | 180 |
| 66 | MSA-C | 56.4 | F | 1.7 | 2 | 3 | 44 | 188 |
| 67 | MSA-C | 68.3 | M | 1.4 | 8 | 6 | 44 | 180 |
| 68 | MSA-P | 58.3 | M | 2.4 | 4 | 4 | 44 | 180 |
| 69 | PD | 52.1 | F | 1.3 | 3 | 2 | 44 | 180 |
| 70 | MSA-C | 62.8 | M | 4.0 | 3 | 7 | 44 | 184 |
| 71 | MSA-P | 63.9 | F | 2.0 | 5 | 7 | 44 | 180 |
| 72 | MSA-C | 54.8 | F | 0.7 | 3 | 4 | 44 | 180 |
| 73 | MSA-C | 74.8 | F | 1.3 | 3 | 6 | 44 | 184 |
| 74 | MSA-P | 67.1 | F | 1.3 | 2 | 4 | 44 | 180 |
| 75 | MSA-P | 74.7 | M | 3.4 | 4 | 4 | 44 | 180 |
| 76 | MSA-P | 72.2 | F | 1.7 | 3 | 6 | 44 | 180 |
| 77 | PD | 73.4 | F | 8.4 | 6 | 5 | 44 | 188 |
| 78 | PD | 67.0 | F | 5.4 | 7 | 2 | 44 | 180 |
| 79 | PD | 72.3 | M | 6.4 | 4 | 1 | 28 | 116 |
| 80 | MSA-P | 71.0 | F | 2.9 | 5 | 7 | 44 | 180 |
| 81 | MSA-C | 71.2 | M | 5.4 | 2 | 9 | 44 | 188 |
| 82 | MSA-P | 74.7 | F | 1.3 | 5 | 7 | 44 | 184 |
| 83 | PD | 63.0 | F | 6.0 | 4 | 3 | 44 | 180 |
| 84 | PD | 67.0 | F | 4.5 | 8 | 2 | 44 | 180 |
| 85 | PD | 68.0 | M | 1.6 | 7 | 2 | 44 | 180 |
| 86 | MSA-C | 69.1 | M | 5.2 | 3 | 9 | 44 | 180 |
| 87 | HC | 51.3 | M | - | 3 | 13 | 44 | 184 |
| 88 | HC | 56.0 | F | - | 3 | 2 | 44 | 180 |
| 89 | HC | 58.5 | M | - | 8 | 3 | 39 | 172 |
| 90 | HC | 55.2 | F | - | 2 | 3 | 44 | 180 |
| 91 | HC | 62.0 | M | - | 3 | 11 | 41 | 176 |
| 92 | HC | 61.9 | F | - | 6 | 3 | 44 | 180 |
| 93 | HC | 60.2 | F | - | 4 | 2 | 44 | 192 |
| 94 | HC | 80.2 | M | - | 15 | 1 | 44 | 184 |
| 95 | HC | 67.5 | F | - | 2 | 2 | 44 | 184 |
| 96 | HC | 70.4 | M | - | 10 | 4 | 44 | 188 |
| 97 | MSA-P | 61.7 | F | 3.2 | 5 | 2 | 44 | 184 |
| 98 | PD | 70.1 | F | 5.0 | 7 | 5 | 44 | 180 |
| 99 | PD | 68.5 | M | 4.9 | 4 | 7 | 44 | 180 |
| 100 | MSA-C | 43.3 | M | 2.5 | 9 | 3 | 44 | 180 |
| 101 | HC | 64.9 | M | - | 4 | 4 | 36 | 156 |
| 102 | HC | 63.7 | M | - | 7 | 1 | 40 | 172 |

# 2 Supplementary Figures

**Supplementary Figure S1. Illustration of masking empirical mode decomposition (EMD).
(A)** Comparison of the original EMD and the masking EMD on a source-reconstructed signal, sampled at 256Hz and with a duration of 8s. The signal is standardized (divided by its standard deviation) to become dimensionless. Both methods decompose the signal to several intrinsic mode functions (IMFs) with fast to slow oscillations. Note that the alpha oscillation is split to IMF 3 and IMF 4 in the original EMD due to the mode mixing problem (see the gray arrow). **(B)** Comparison of the marginal Hilbert spectrum of individual IMFs obtained via the original EMD and the masking EMD. The x-axis is shown on the logarithmic scale.

**Supplementary Figure S2. Quality control of the IMFs from masking EMD.**

**(A)** The marginal Hilbert spectrum (power-weighted histogram of instantaneous frequency) of each IMF. Individual spectral lines of 102 participants × 100 cortical ROIs were plotted, with their mean shown as black dashed lines. The marginal Hilbert spectrum were normalized by the total power of all IMFs within each cortical ROI and each participant, such that the area under the curves of all IMFs summed to 100%. The center frequency, half-power bandwidth (a.k.a. full width at half maximum), and normalized total power of each IMF were calculated and shown as mean ± standard deviation (SD) (*n* = 10200). **(B)** The Fourier power spectrum of each IMF.
